# Cross-Sensor Consistency-Guided Dual-Spectrum Fusion for Offshore Wind Turbine Blade Defect Diagnosis and Risk Grading

**DOI:** 10.3390/s26123878

**Published:** 2026-06-18

**Authors:** Yukun Wang, Chenhao Sun, Ruifeng Liao, Lijun Luo, Jiefeng Duan

**Affiliations:** 1 State Key Laboratory of Disaster Prevention and Reduction for Power Grid, Changsha University of Science and Technology, Changsha 410114, China; m17662727518@163.com; 2 State Grid Zhejiang Electric Power Co., Ltd., Lishui Power Supply Company, Lishui 323000, China; m13735920570@163.com; 3 Hunan Wuling Electric Power Technology Co., Ltd., Changsha 410205, China; luo_llj@wldl.com.cn (L.L.); 18833075477@163.com (J.D.)

**Keywords:** offshore wind turbine blade, defect diagnosis, dual-spectrum imaging, cross-sensor consistency, risk grading

## Abstract

Offshore wind turbine blades are chronically exposed to complex marine environments with high humidity, salt spray, strong wind, waves, and intense radiation. Under such conditions, blade defects often exhibit small sizes, weak visual features, and heterogeneous visible infrared manifestations. Conventional single-sensor monitoring and empirically weighted fusion methods are insufficient for reliable defect diagnosis and risk grading. To address this problem, this paper proposes a cross-sensor consistency-guided dual-spectrum fusion framework, termed CG-DSF, for offshore wind turbine blade defect diagnosis and risk assessment. First, visible-light images and infrared thermal images are acquired by UAV-mounted imaging sensors, and sensor-specific branches are constructed to extract surface structural features and thermal anomaly responses. Second, visible and infrared features are aligned at the feature token level, and cross-sensor evidence is evaluated for spatial consistency, diagnostic semantic consistency, and anomaly consistency. A reliability-aware fusion strategy is then used to suppress low-quality or conflicting observations and construct a unified defect representation. Finally, a series of representative simulation case studies are carried out to comprehensively assess the overall performance and practical applicability of the constructed model. Experimental results reveal that the proposed framework possesses evident advantages in blade defect identification for offshore wind turbines, offering a feasible solution for advancing proactive and intelligent condition-based operation and maintenance of offshore wind assets in complex marine environments.

## 1. Introduction

### 1.1. Research Background

With the continuous expansion of renewable energy installed capacity, offshore wind power has become an important component in the construction of new power systems and the transformation of the energy structure. Compared with onshore wind power, offshore wind farms operate for long periods in complex marine environments characterized by high humidity, high salt spray, strong wind and waves, and intense ultraviolet radiation. As key components directly subjected to aerodynamic loads and environmental erosion in wind turbines, wind turbine blades are highly susceptible to defects such as surface cracks, leading edge erosion, coating peeling, and material fatigue degradation under such conditions [1,2]. If blade defects are not detected and addressed in a timely manner, the wind energy capture efficiency and power generation performance of the unit may be reduced, and blade structural failure, shutdown maintenance, or even major safety accidents may be induced [3]. Therefore, efficient detection, accurate diagnosis, and early risk warning of offshore wind turbine blade defects have become important research topics in intelligent operation and maintenance of offshore wind power [4].

Traditional wind turbine blade inspection mainly relies on manual visual inspection, telescope-based observation, shutdown, and close-range inspection. These methods are characterized by low detection efficiency, high cost, and long inspection cycles, and are difficult to satisfy the refined operation and maintenance requirements of large-scale offshore wind farms [5]. In recent years, with the rapid development of unmanned aerial vehicle platforms, visible-light imaging sensors, infrared thermal imaging sensors, and deep learning techniques, intelligent wind turbine blade inspection based on multisource sensing information has gradually become a research focus [6,7]. Compared with traditional inspection methods, unmanned aerial vehicle inspection has the advantages of strong mobility, wide coverage, and low operational risk, and blade surface images and thermal anomaly information can be acquired [8].

However, due to the large size, complex curved surface structure, and small defect scale of offshore wind turbine blades, as well as the complex backgrounds and harsh environments during inspection, existing methods still face insufficient detection stability in complex marine environments. Meanwhile, a single sensor usually reflects only one type of local information related to blade health status. For example, visible-light images mainly characterize surface morphology, whereas infrared thermal images mainly characterize thermal anomaly responses. When used separately, neither can comprehensively describe the true state of blade defects. Existing fixed-weight or simple concatenation-based multi-sensor fusion methods are also insufficient for fully considering data quality differences and physical correlations among different sensors. Moreover, most studies remain limited to defect detection and classification, with insufficient analysis of defect severity and operational risk.

### 1.2. Related Work

In recent years, with the continuous growth of offshore wind power operation and maintenance requirements, wind turbine blade defect detection has gradually evolved from traditional manual inspection and offline nondestructive testing into an intelligent condition perception and fault diagnosis problem based on multisource sensing data. Extensive studies have been conducted on visible-light images, infrared thermal images, fiber optic strain, acoustic signals, vibration responses, and other sensing modalities, providing an important foundation for wind turbine blade defect detection [9,10]. However, offshore wind turbine blade inspection scenarios are commonly characterized by small defect scales, complex backgrounds, unstable sensing data quality, and severe environmental interference. Consequently, existing methods still face considerable challenges in complex engineering applications.

Existing studies are mainly concentrated in three categories.

(1) Defect detection methods based on a single sensor

This category of methods mainly relies on one type of sensor data for blade defect identification and diagnosis. Methods based on visible-light imaging sensors usually acquire blade surface images through unmanned aerial vehicles. Early studies mostly relied on edge detection, texture analysis, morphological processing, and manually designed features for defect identification. With the development of deep learning, methods based on convolutional neural networks, Transformer, and object detection networks have gradually become mainstream, and the detection accuracy of surface defects such as cracks, erosion, and coating peeling can be improved to a certain extent [11,12,13]. Recent studies have also shown that lightweight Transformer structures, feature fusion mechanisms, and global–local representation learning can improve fault diagnosis performance in rotating machinery and related intelligent diagnosis tasks, which provides useful references for designing efficient feature extraction and fusion modules in blade inspection scenarios [14]. In addition to visual detection, infrared thermal imaging sensors can assist in identifying local thermal anomaly regions through variations in the temperature field, which is of certain value for detecting surface damage, subsurface defects, and fatigue damage [15,16]. Acoustic sensors can also be used to perceive changes in acoustic propagation characteristics caused by blade structural damage [17].

Although the above single-sensor methods have achieved certain progress in wind turbine blade defect detection, their limitations remain evident. First, visible-light images mainly reflect the surface appearance information of blades and are susceptible to complex background interference, with insufficient perception capability for hidden defects such as internal debonding, delamination, and early material degradation. Second, infrared thermal images can provide thermal anomaly information, but the detection performance is easily affected by solar radiation, ambient temperature differences, and thermal drift, and misjudgment may occur when they are used alone. Finally, although sensing methods based on fiber optic strain, acoustic signals, and vibration responses can reflect changes in blade structural responses, they are usually sensitive to sensor deployment locations, signal noise, operating conditions, and long-term stability and are difficult to directly satisfy the requirements of large-scale unmanned aerial vehicle inspection scenarios. Therefore, single-sensor methods can usually characterize only one type of response feature of blade defects, and a comprehensive and stable representation of the true health state of wind turbine blades in complex marine environments remains difficult.

(2) Defect inspection methods based on multi-sensor fusion.

To overcome the incomplete information representation of single-sensor methods, multisource sensing information, such as visible-light images and infrared thermal images, has been increasingly fused in recent studies to improve the accuracy and robustness of wind turbine blade defect diagnosis [18,19]. Visible-light images can reflect surface structural damage, such as blade surface cracks, leading edge erosion, and coating peeling, whereas infrared thermal images can provide local thermal anomaly and suspected internal damage information from the perspective of temperature response. Blade states are, therefore, described from both appearance morphology and thermal response, showing strong complementarity [20].

Although visible and infrared observations are complementary, how to fuse them remains a critical issue. Many existing multi-sensor inspection methods adopt feature concatenation, score-level averaging, empirically fixed weighting, or attention-based interaction to combine heterogeneous information. These strategies can improve the amount of available diagnostic information, but they usually treat multimodal fusion as a feature aggregation problem rather than an evidence verification problem. In practical offshore inspection, the visible branch may respond strongly to surface texture changes caused by cracks, erosion, or coating damage, whereas the infrared branch may respond to thermal diffusion, local hot spots, or background temperature fluctuations. These responses may not always be spatially coincident or diagnostically consistent. For example, a visually evident surface scratch may have no corresponding thermal anomaly, while a local thermal discontinuity may appear in a region with only weak visual evidence. If the two types of features are directly concatenated or weighted without checking their relationship, unreliable sensor evidence, background interference, or mutually conflicting responses may be propagated into the final diagnosis.

Conventional attention-based fusion can assign larger weights to salient feature channels or regions, but the learned attention weights are usually implicit and do not explicitly answer three diagnostic questions: whether the two sensors focus on the same defect-related region, whether they support the same defect category or abnormal state, and whether their abnormal response intensities are mutually consistent. Therefore, the key limitation of existing fusion strategies is not only the lack of multimodal information but also the lack of explicit cross-sensor consistency modeling before fusion.

(3) Risk assessment methods for operation and maintenance decision making.

Most existing studies on wind turbine blade defect detection mainly focus on metrics such as detection accuracy, recall, classification accuracy, and model inference efficiency, while insufficient attention has been paid to defect severity assessment and risk-level classification [21]. However, in practical offshore wind farm inspection, inspectors are required not only to determine whether defects exist on blades, but also to further obtain information such as defect location, defect area, crack length, erosion degree, and thermal anomaly intensity, so as to determine whether the defect requires focused attention. Some studies have begun to use multispectral unmanned aerial vehicle data fusion and fuzzy logic for wind turbine equipment defect criticality assessment, indicating that the transition from defect detection to severity or risk-level assessment has become a direction worthy of attention [22,23]. In a broader intelligent fault diagnosis context, neural network-based fault estimation and reinforcement iterative learning schemes have also been explored to improve fault estimation accuracy and robustness under uncertain operating conditions, indicating that learning-based fault diagnosis is gradually extending from state recognition to reliability-oriented decision support [24].

At present, many methods still remain at the level of defect localization or type identification, and their outputs are usually detection boxes, class labels, or classification probabilities, which can hardly directly reflect the impact of defects on blade health status. For the same type of blade defect, its risk degree is often affected by factors such as defect size, defect location, thermal anomaly response, and local propagation tendency. If only defect categories are provided, the requirement for graded judgment in practical inspection can hardly be satisfied. Therefore, further extension from “defect identification” to “severity assessment and risk level analysis”, followed by reference support for subsequent maintenance judgment, has become a direction that deserves further attention in intelligent wind turbine blade inspection research.

In summary, existing studies have provided important foundations for offshore wind turbine blade inspection, including single-sensor defect recognition, multi-sensor information fusion, and preliminary risk-oriented assessment. However, three limitations remain insufficiently addressed. First, single-sensor methods are vulnerable to complex offshore inspection conditions because visible images and infrared thermal images describe different but incomplete aspects of blade damage. Second, most existing multi-sensor fusion strategies, such as direct feature concatenation, empirically fixed weighting, or conventional attention-based fusion, mainly increase the feature richness but do not explicitly determine whether the evidence from different sensors supports the same defect region, the same diagnostic tendency, or a similar abnormal degree. As a result, unreliable or conflicting sensor evidence may be introduced into the final diagnosis under illumination degradation, sea fog, thermal drift, motion blur, or local sensor mismatch. Third, many existing studies still focus on defect detection or category classification, while the connection between defect evidence, severity assessment, and inspection priority remains insufficient.

### 1.3. Statement and Contributions

To address these gaps, this study proposes a cross-sensor consistency-guided dual-spectrum fusion framework, termed CG-DSF, for offshore wind turbine blade defect diagnosis and risk grading. Different from conventional fusion methods that combine visible and infrared features directly, CG-DSF first aligns dual-spectrum representations at the feature level and then evaluates cross-sensor evidence for spatial consistency, diagnostic semantic consistency, and anomaly consistency. On this basis, reliability-aware fusion is performed to suppress low-quality or conflicting observations and to construct a unified defect representation. The proposed framework further converts defect localization and classification results into severity-oriented risk levels, so that the output can provide more direct support for inspection review and maintenance prioritization.

**Figure 1:**
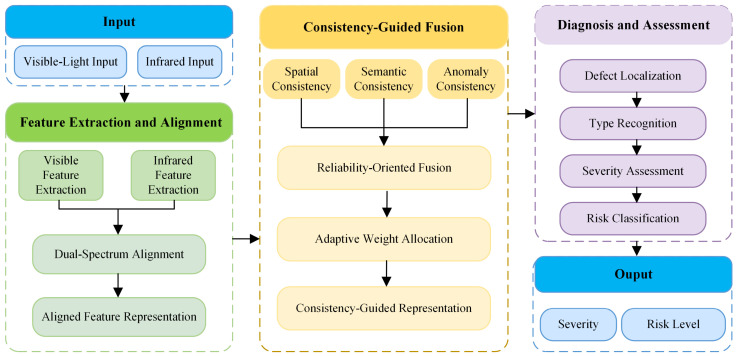
Overall workflow of the proposed CG-DSF framework.

As shown in Figure 1, the overall workflow of CG DSF consists of three main stages.

The first stage is dual-spectrum imaging sensor feature extraction and alignment. Considering the different imaging mechanisms and distinct feature distributions of visible-light images and infrared thermal images, a visible-light branch and an infrared thermal imaging branch are constructed separately for independent feature modeling of the two types of sensing data. The visible-light branch focuses on structural features such as blade surface texture, defect boundaries, crack morphology, and erosion regions. The infrared branch focuses on thermal features such as temperature gradients, local hot spots, thermal response continuity, and abnormal thermal distributions. On this basis, feature-level alignment is performed for the two types of sensing representations, so that comparability can be established at the blade region and defect candidate region levels.

The second stage is cross-sensor consistency guided fusion. This stage constitutes the core of CG-DSF. In this paper, the correlation between visible-light images and infrared thermal images is characterized from three aspects: spatial consistency, diagnostic semantic consistency, and anomaly consistency. Spatial consistency is used to determine whether regional correspondence exists between the surface defect regions attended to by the visible-light branch and the thermal anomaly regions responded to by the infrared branch. Diagnostic semantic consistency is used to determine whether the two types of sensors provide consistent discrimination results for the defect categories or abnormal states. Anomaly consistency is used to measure whether the significance of surface structural damage matches the intensity of thermal anomaly responses. On this basis, the two types of features are adaptively fused according to consistency relationships and differences in sensing data reliability. Interference from low-quality or mutually conflicting sensing information is suppressed, and a unified defect representation for blade defect diagnosis is formed.

The third stage is defect diagnosis and severity assessment. The fused unified defect representation contains both surface structural damage information provided by the visible-light branch and thermal anomaly response information provided by the infrared branch. Based on this representation, blade defect localization and type identification are first performed to obtain defect regions and the corresponding category judgments. Subsequently, defect morphology features, thermal anomaly intensity, and cross-sensor consistency information are further integrated to assess the defect severity and classify the corresponding risk level.

The main contributions of this paper are summarized as follows:

(1) A dual-spectrum imaging sensor framework is developed for offshore wind turbine blade defect diagnosis. The proposed framework jointly models RGB-based surface structural evidence and infrared thermal anomaly evidence through two sensor-specific branches. Compared with single-sensor diagnosis, this design allows the model to capture both visible morphology, such as cracks, leading-edge erosion, and coating damage, and thermal responses, such as local hot spots, temperature gradients, and thermal discontinuities, which improves the completeness of blade health-state representation under complex offshore inspection conditions;
(2) A cross-sensor consistency-guided fusion strategy is proposed to distinguish reliable dual-sensor support from unreliable or conflicting observations. Different from direct feature concatenation, fixed-weight fusion, or conventional attention-based fusion, the proposed method explicitly evaluates visible–infrared evidence from three complementary perspectives: spatial consistency, diagnostic semantic consistency, and anomaly consistency. These consistency cues are further combined with sensor reliability estimation to regulate the contribution of each modality, so that low-quality or inconsistent evidence can be suppressed before constructing the unified defect representation;
(3) A severity-oriented risk grading mechanism is designed to connect defect diagnosis with inspection-priority decision making. Instead of treating classification confidence as defect severity, the proposed mechanism integrates defect morphology, thermal anomaly intensity, diagnostic confidence, and cross-sensor consistency evidence to generate ordinal risk levels and continuous risk scores. This design allows the model to provide not only defect localization and category identification, but also interpretable low-, medium-, and high-risk outputs for maintenance review and priority inspection.

## 2. Methodology

### 2.1. Visible–Infrared Bimodal Sensor Feature Extraction

Visible-light images and infrared thermal images constitute two important types of imaging sensing information in offshore wind turbine blade inspection, through which the same blade state is described from different observational perspectives [25]. Visible-light images mainly provide information related to the appearance and structural characteristics of blades, whereas infrared thermal images mainly provide information related to thermal responses. Based on dual-spectrum imaging sensor inputs, a dual-branch feature extraction structure is adopted in this paper to process visible-light images and infrared thermal images separately. The visible-light branch is used to form the visual representation of the blade appearance, while the infrared branch is used to form the infrared representation of the blade thermal response. After independent encoding is completed by the two branches, a unified comparison basis is established through cross-sensor feature alignment, so that the relationships between the two types of sensing information can be analyzed by the subsequent fusion module under relatively consistent feature scales and regional semantics.

#### 2.1.1. RGB Visual Feature Extraction

**Figure 2:**
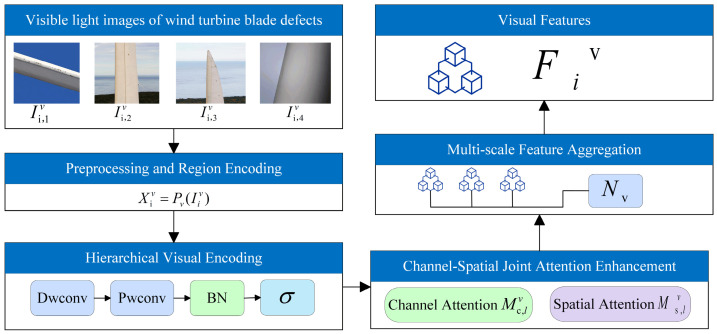
RGB visible feature extraction process.

Visible-light images are the most direct source of appearance and structural observation in offshore wind turbine blade inspection and are mainly used to capture apparent damage information such as surface texture variations, edge contours, crack trajectories, leading edge erosion, coating peeling, and local contamination [26]. In offshore inspection scenarios, visible-light images are usually affected by illumination variation, sea fog occlusion, motion blur, specular reflection from curved blade surfaces, and complex sea sky backgrounds. As a result, defect regions often appear as small-scale, weak boundary, and low contrast targets. Therefore, visible-light images are not regarded merely as ordinary RGB inputs in this paper. Instead, they are treated as surface structural damage sensing information, with an emphasis placed on the extraction of multiscale visual features that can reflect blade defect morphology. The visible-light visual feature extraction process is shown in Figure 2:

The visible-light images used in this paper were acquired by an optical RGB imaging sensor mounted on an unmanned aerial vehicle, based on in-service wind turbine blade data from the public dataset of the AQUADA-GO project.

Compared with shutdown close-range imaging, this type of data is closer to practical inspection scenarios, where blade regions may exhibit pose variation, scale variation, and background interference. Therefore, before being fed into the visual feature extraction network, the visible-light images are first subjected to scale normalization, blade region cropping, and pixel normalization, so that the input distribution shifts caused by differences in imaging distance, field of view, and illumination conditions among different video frames can be reduced. Let the visible-light image of the i-th sample be denoted as $I_{i}^{v}$. The preprocessed input can be expressed as:

(1) $$X_{i}^{v}=P_{v}\left(I_{i}^{v}\right)$$

where $P_{v}$ denotes the visible-light image preprocessing operation, including size normalization, pixel standardization, and blade region enhancement, and $X_{i}^{v}$ denotes the standardized RGB image input to the visual encoder.

To address the significant scale variation and complex boundary morphology of blade surface defects, a hierarchical visible-light visual encoder is constructed in this paper for multiscale feature extraction from $X_{i}^{v}$. The encoder consists of several convolutional feature extraction units. Low-level visual information, such as edges, textures, local grayscale variations, and crack contours, is mainly extracted by the shallow layers, while high-level semantic information related to erosion regions, peeling morphology, and defect categories is further extracted by the deeper layers. Considering the requirements of unmanned aerial vehicle inspection tasks for both model response speed and feature representation capability, lightweight convolutional units are adopted as the basic feature extraction structure in the visual branch. Standard convolution is decomposed into depthwise convolution and pointwise convolution to reduce the number of parameters and computational complexity [27]. The visual feature extraction process at the l-th layer can be expressed as:

(2) $$X_{i,l}^{v}=\phi_{l}^{v}\left(X_{i,l-1}^{v}\right)=\sigma (BN(PWConv(DWConv(X_{i,l-1}^{v}))))$$

where $X_{i,l}^{v}$ denotes the visible-light feature at the l-th layer, DWConv denotes depthwise convolution, PWConv denotes 1×1 pointwise convolution, BN denotes batch normalization, and σ denotes the nonlinear activation function. With this structure, hierarchical representation of fine grained blade surface defect features can be achieved by the visible-light branch with relatively low computational overhead.

Since surface defects on wind turbine blades usually occupy only small regions in images and are easily disturbed by background textures, illumination variations, and specular reflection on blade surfaces, a channel spatial joint attention mechanism is further introduced into the visible-light visual encoder to adaptively enhance the key defect features [28]. Specifically, channel attention is used to model the importance of different feature channels for defect identification, with effective responses such as crack edges, peeling textures, and erosion morphology strengthened. Spatial attention is used to highlight local regions that are more likely to contain defects, while interference from sea sky backgrounds, blade shadows, and irrelevant textures is suppressed. For the l-th layer feature $X_{i,l}^{v}$, its channel attention weight can be expressed as:

(3) $$M_{c,l}^{v}=\delta (MLP(AvgPool(X_{i,l}^{v}))+MLP(MaxPool(X_{i,l}^{v})))$$

The spatial attention weight can be expressed as:

(4) $$M_{s,l}^{v}=\delta (f^{7\times 7}\left(\left[{AvgPool}_{c}\left(M_{c,l}^{v}⊙X_{i,l}^{v}\right);{MaxPool}_{c}\left(M_{c,l}^{v}⊙X_{i,l}^{v}\right)\right]\right))$$

where δ denotes the Sigmoid activation function, MLP denotes the shared multilayer perceptron, $f^{7\times 7}$ denotes the spatial convolution operation with a kernel size of 7×7, and ⊙ denotes element-wise multiplication. The visible-light feature after attention enhancement is expressed as:

(5) $${\hat{X}}_{i,l}^{v}=M_{s,l}^{v}⊙M_{c,l}^{v}⊙X_{i,l}^{v}$$

Through this process, more discriminative defect responses can be selected along the channel dimension, while potential defect regions on the blade surface can be preferentially emphasized along the spatial dimension. As a result, the distinguishability of defects with weak features is improved.

To accommodate different defect types, including small-scale cracks and large-scale erosion or peeling, multilevel visual features are further aggregated across scales in this paper. Shallow enhanced features retain higher spatial resolution and are suitable for describing crack boundaries and fine-grained textures, whereas deep enhanced features provide stronger semantic representation capability and are suitable for characterizing defect categories and overall morphology. In this paper, enhanced features at different scales are uniformly mapped to the same channel dimension, and the final visible-light representation is obtained through feature pyramid-based aggregation:

(6) $$F_{i}^{v}=N_{v}\left({\hat{X}}_{i,3}^{v},{\hat{X}}_{i,4}^{v},{\hat{X}}_{i,5}^{v}\right)$$

where $N_{v}$ denotes the multiscale visual feature aggregation operation, ${\hat{X}}_{i,3}^{v},{\hat{X}}_{i,4}^{v},{\hat{X}}_{i,5}^{v}$ denote the attention-enhanced features at different scales, respectively, and $F_{i}^{v}$ denotes the visual feature output by the visible-light branch. This feature contains local boundary information, texture variation information, and high-level semantic information of blade surface defects, and can provide a comparable visual basis for subsequent cross-sensor feature alignment and consistency guided fusion.

It should be noted that the visible-light branch is not intended to independently complete the final defect diagnosis, but to provide a reliable representation of surface structural damage for dual-spectrum fusion. Therefore, the $F_{i}^{v}$ output from the visible-light branch is used as the input to the subsequent cross-sensor feature alignment module in this paper.

#### 2.1.2. Infrared Thermal Feature Extraction

**Figure 3:**
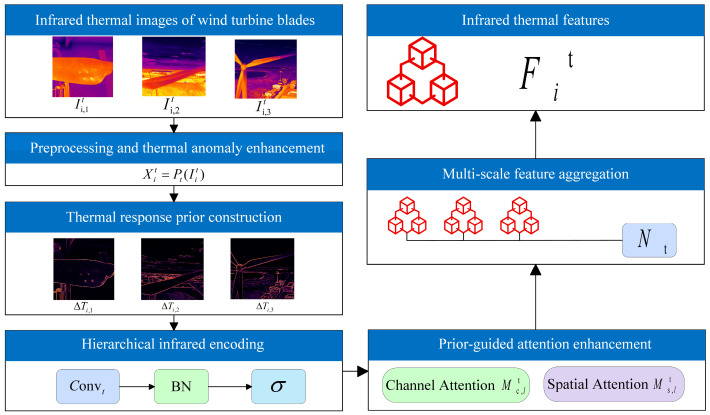
Infrared thermal feature extraction process.

Infrared thermal images are used to characterize the thermal abnormal responses of blade defects and serve as an important complement to visible-light images [29]. Different from visible-light images, which mainly reflect surface textures, crack boundaries, and erosion morphology, infrared thermal images record the temperature distribution and thermal radiation differences on blade surfaces, through which local hot spots, abrupt temperature gradient changes, discontinuous thermal responses, and abnormal manifestations of suspected internal debonding or material degradation regions can be revealed [30]. Existing studies have shown that, under appropriate illumination conditions, infrared thermal images of wind turbine blades acquired by using solar irradiation as a passive thermal excitation source can identify thermal abnormal responses caused by damage such as wear and cracks within a certain range. Meanwhile, under blade curved surface structures, nonuniform thermal excitation, and low clarity conditions, infrared images are confronted with problems such as blurred details and insufficient contrast. Therefore, image enhancement and deep feature extraction methods are required to improve the recognizability of abnormal regions. The infrared thermal image feature extraction process is shown in Figure 3:

The infrared thermal images used in this paper were acquired by an infrared thermal imaging sensor mounted on an unmanned aerial vehicle, with data collected from 22 operating wind turbines in the public dataset of the AQUADA-GO project.

Considering that blade surface temperature during offshore inspection can be affected by solar irradiation, sea wind cooling, sea fog occlusion, background temperature differences, and sensor thermal drift, temperature normalization, background suppression, and thermal anomaly enhancement are first performed on the infrared thermal images in this paper, so that better comparability of the thermal response distributions across different frames can be obtained. Let the infrared thermal image of the i-th sample be denoted as $I_{i}^{t}$ Its standardized input is expressed as:

(7) $$X_{i}^{t}=P_{t}\left(I_{i}^{t}\right)$$

where $P_{t}$ denotes the infrared thermal image preprocessing function, which mainly includes size normalization, temperature response normalization, noise suppression, background thermal response correction, and blade region enhancement. $X_{i}^{t}$ denotes the standardized thermal image input to the infrared thermal imaging encoder.

Considering that overall temperature shifts may exist among different video frames in offshore inspection environments, a local background-based relative thermal anomaly normalization strategy is further adopted in this paper, through which the original temperature response is converted into a relative thermal anomaly response. For the temperature response $T_{i}(p)$ at pixel position p in the infrared thermal image, its relative thermal anomaly response is defined as:

(8) $$∆T_{i}\left(p\right)=\frac{T_{i}(p)-\mu_{i}^{b}}{\sigma_{i}^{b}+\varepsilon }$$

where $\mu_{i}^{b}$ and $\sigma_{i}^{b}$ denote the mean temperature and standard deviation of the blade background region in the current infrared frame, respectively, and ε denotes a stabilization term used to avoid a zero denominator. Through this processing, global thermal offsets caused by environmental temperature, irradiation intensity, and sensor drift can be reduced, and greater attention can be assigned to local thermal anomalies in defect regions relative to the blade background.

On this basis, a thermal response prior map is constructed to enhance the perception capability of the infrared branch for thermal anomaly boundaries and abrupt local temperature changes. Specifically, the prior thermal response is composed of the relative thermal anomaly amplitude and its temperature gradients in the horizontal and vertical directions:

(9) $$R_{i}^{t}=Concat(∆T_{i},\left|\nabla_{x}\Delta T_{i}\right|,\left|\nabla_{y}\Delta T_{i}\right|)$$

where $|\nabla_{x}\Delta T_{i}|$ and $|\nabla_{y}\Delta T_{i}|$ mid denote the gradient intensities of the relative thermal anomaly response in the horizontal and vertical directions, respectively, and Concat denotes the channel concatenation operation. Compared with the use of only the original infrared grayscale response, $R_{i}^{t}$ contains both thermal anomaly intensity and thermal boundary variation information, which is beneficial for highlighting local hot spots, abrupt thermal gradient changes, and thermal response discontinuity regions. To improve the adaptability of the infrared branch to complex offshore inspection environments, data augmentation strategies, including temperature perturbation, random cropping, scale transformation, slight rotation, local noise injection, and thermal contrast perturbation, are further introduced during training to simulate infrared imaging variations under different imaging distances, blade poses, background temperature differences, and sensor noise conditions.

In the infrared thermal feature extraction stage, an infrared thermal imaging encoder is constructed in this paper for hierarchical feature modeling of the standardized thermal image $X_{i}^{t}$. Different from the visible-light branch, which focuses on blade surface texture, edges, and crack contours, the infrared branch places greater emphasis on temperature distribution patterns, local hot spot morphology, thermal gradient variations, and abnormal thermal response continuity. Therefore, a hierarchical convolutional structure is adopted in the infrared encoder to progressively extract thermal features from shallow to deep layers. The infrared feature extraction process at the l-th layer is expressed as:

(10) $$X_{i,l}^{t}=\phi_{l}^{t}\left(X_{i,l-1}^{t}\right)=\sigma (BN({Conv}_{t}\left(X_{i,l-1}^{t}\right)))$$

where $X_{i,l}^{t}$ denotes the infrared thermal feature at the l-th layer, $\phi_{l}^{t}$ denotes the infrared thermal imaging feature extraction unit, ${Conv}_{t}$ denotes the convolution operation in the infrared branch, BN denotes batch normalization, and σ denotes the nonlinear activation function. Through this process, thermal boundaries, hot spot morphology, temperature gradients, and region-level abnormal thermal distribution semantics can be progressively extracted from infrared thermal images.

To avoid a simple formal repetition between the infrared branch and the visible-light branch, a thermal response prior guided attention enhancement mechanism is introduced into the infrared feature extraction process. The basic idea of channel spatial joint attention is retained in this mechanism, but its spatial attention is no longer generated only from the infrared feature itself. Instead, relative thermal anomaly and temperature gradient information are further incorporated, so that greater attention is assigned to abnormal regions with explicit thermal significance. First, infrared channel attention is used to select the effective feature channels related to local hot spots, temperature gradients, and abnormal thermal distributions, and its weight is expressed as:

(11) $$M_{c,l}^{t}=\delta ({MLP}_{t}(AvgPool\left(X_{i,l}^{t}\right))+{MLP}_{t}(MaxPool\left(X_{i,l}^{t}\right)))$$

where δ denotes the Sigmoid activation function, ${MLP}_{t}$ denotes the independent shared multilayer perceptron in the infrared branch, and AvgPool and MaxPool denote the average pooling and maximum pooling operations, respectively. Subsequently, the prior thermal response $R_{i}^{t}$ is mapped to the feature scale of the l-th layer:

(12) $$R_{i,l}^{t}=\psi_{l}\left(R_{i}^{t}\right)$$

where $\psi_{l}$ denotes the scale mapping function, which is used to ensure spatial size consistency between the prior thermal response and the infrared feature $X_{i,l}^{t}$. On this basis, the infrared spatial attention is expressed as:

(13) $$M_{s,l}^{t}=\delta \left(f_{t}^{7\times 7}\left(\left[{AvgPool}_{c}\left(M_{c,l}^{t}⊙X_{i,l}^{t}\right);{MaxPool}_{c}\left(M_{c,l}^{t}⊙X_{i,l}^{t}\right);R_{i,l}^{t}\right]\right)\right)$$

where $f_{t}^{7\times 7}$ denotes the spatial convolution operation of the infrared branch, and ⊙ denotes element-wise multiplication. Compared with conventional spatial attention, temperature anomaly amplitude and thermal gradient variations are explicitly incorporated into the spatial attention calculation process in this design. Thus, regions with thermal anomaly significance can still be preferentially emphasized by the infrared branch under conditions of blurred thermal boundaries, weak thermal contrast, or strong interference from background temperature differences. The infrared feature after prior thermal response guided attention enhancement is expressed as:

(14) $${\hat{X}}_{i,l}^{t}=M_{s,l}^{t}⊙M_{c,l}^{t}⊙X_{i,l}^{t}$$

Finally, multiscale aggregation is performed on infrared attention-enhanced features at different levels to form the final feature representation of the infrared thermal image branch. Shallow infrared features retain higher spatial resolution and are suitable for describing local hot spot boundaries, temperature abrupt changes, and small-scale thermal anomalies. Deep infrared features possess stronger region-level representation capability and are suitable for characterizing thermal response continuity, abnormal thermal distribution patterns, and the thermal semantics of suspected damage regions. The infrared multiscale feature aggregation process is expressed as:

(15) $$F_{i}^{t}=N_{t}\left({\hat{X}}_{i,3}^{t},{\hat{X}}_{i,4}^{t},{\hat{X}}_{i,5}^{t}\right)$$

where $N_{t}$ denotes the infrared thermal feature aggregation function, ${\hat{X}}_{i,3}^{t}$, ${\hat{X}}_{i,4}^{t}$, and ${\hat{X}}_{i,5}^{t}$ denote the infrared attention-enhanced features at different scales, respectively, and $F_{i}^{t}$ denotes the thermal anomaly feature output by the infrared thermal image branch. This feature comprehensively contains the temperature gradient, local hot spot, thermal response continuity, and abnormal thermal distribution information.

It should be noted that visible-light attention mainly enhances surface structural damage features according to the texture, edges, and spatial saliency. In contrast, infrared attention further incorporates relative thermal anomaly and temperature gradient information as thermal response priors, so that greater emphasis is placed by spatial attention on local hot spots, thermal boundaries, and abnormal thermal distribution regions. Therefore, the two branches maintain a consistent attention modeling framework in form, while their parameters are independent, their input features are different, and physical constraints specific to infrared thermal imaging sensors are incorporated into the infrared branch.

#### 2.1.3. Cross-Sensor Feature Alignment

After independent encoding by the aforementioned visible-light branch and infrared thermal image branch, the surface appearance side visual feature $F_{i}^{v}$ and the thermal response side infrared feature $F_{i}^{t}$ are obtained by the model, respectively. Although these two types of features correspond to the same inspection sample, they are not naturally consistent in feature map scale and semantic expression because of differences in imaging field of view, spatial resolution, feature channel dimension, and response region range. If $F_{i}^{v}$ and $F_{i}^{t}$ are directly concatenated or weighted, spatially mismatched regions, scale-inconsistent responses, and sensor-specific noise may be jointly input into the fusion module, causing interference with the subsequent cross-sensor relationship modeling. Therefore, before consistency-guided fusion, a cross-sensor feature alignment module is introduced in this paper to map the two types of independent features into a unified comparison space, so that visible-light structural information and infrared thermal anomaly information can be analyzed under the same feature scale and regional index [31].

Let the output feature of the visible-light branch be $F_{i}^{v}\in R^{H_{v}\times W_{v}\times C_{v}}$, and the output feature of the infrared thermal image branch be $F_{i}^{t}\in R^{H_{t}\times W_{t}\times C_{t}}$. Since their spatial sizes and channel dimensions may differ, scale transformation and channel projection are first adopted in this paper for basic feature normalization:

(16) $${\bar{F}}_{i}^{m}=G_{m}\left(F_{i}^{m}\right)$$

where $G_{m}$ denotes the feature normalization mapping of the m-th type of sensor, which consists of spatial resampling and 1×1 convolution or linear projection, with $m\in \left\{v,t\right\}$; ${\bar{F}}_{i}^{m}\in R^{H_{a}\times W_{a}\times d}$ denoting the mapped feature and $H_{a}$ $W_{a}$ and d denoting the unified spatial size and channel dimension, respectively. The purpose of this process is to eliminate the basic differences between the two types of features in size and dimension, without altering the sensor-specific representations already learned by their respective branches.

After basic normalization is completed, the feature maps are converted into region-level feature sequences, so that cross-sensor comparable regional indices can be established. Specifically, Fim is unfolded according to the spatial positions, and positional encoding is added to obtain:

(17) $$Z_{i}^{m}=Flatten\left({\bar{F}}_{i}^{m}\right)+E_{p}^{m}$$

where $Z_{i}^{m}\in R^{L\times d}$, $L=H_{a}W_{a}$, and $E_{p}^{m}$ denotes the positional encoding, which is used to preserve the relative spatial relationships of different regions in the blade image. Unlike the strict registration directly performed at the pixel level, the feature-level regional representation adopted here can maintain regional correspondence between the two sensor features, even under slight viewpoint deviations, local thermal diffusion, and imaging scale variations.

To further reduce the discrepancy between the visible-light features and the infrared thermal features in the representation space, shared regional anchors are introduced in this paper to provide unified indexing for the two types of features. Let the shared regional anchors be denoted as $Q\in R^{K\times d}$, where K denotes the number of aligned region tokens. This anchor set does not belong to any individual sensor but serves as the regional query reference in the unified comparison space. For any sensor branch, its regional alignment weight is defined as:

(18) $$A_{i}^{m}=Softmax\left[\frac{{\left(QW_{q})(Z_{i}^{m}W_{k}^{m}\right)}^{T}}{\sqrt{d}}\right]$$

where $W_{q}$ denotes the shared query mapping matrix, $W_{k}^{m}$ denotes the key mapping matrix of the m-th type of sensor, and $A_{i}^{m}\in R^{K\times L}$ denotes the response relationship between the shared regional anchors and the original feature regions. Subsequently, the aligned sensor feature representation is obtained as:

(19) $${\tilde{F}}_{i}^{m}=A_{i}^{m}\left(Z_{i}^{m}W_{v}^{m}\right)$$

where $W_{v}^{m}$ denotes the value mapping matrix of the m-th type of sensor, and ${\tilde{F}}_{i}^{m}\in R^{K\times d}$ denotes the feature mapped to the unified regional index. After this processing, the visible-light features and infrared thermal features share the same number of tokens and the same feature dimension, and each region token corresponds to the same anchor position in the unified comparison space.

The aligned ${\tilde{F}}_{i}^{v}$ and ${\tilde{F}}_{i}^{t}$ still retain the independent information sources of the visible-light branch and the infrared branch, respectively, while consistency is maintained in regional indices, channel dimensions, and representation scales. The two features characterize the blade appearance, structural information, and thermal anomaly response information, respectively, providing a basis for a subsequent analysis of the relationships between the dual-spectrum sensing information.

It should be noted that the proposed alignment is performed at the feature-token level rather than at the original pixel level. If the number of feature regions is N, the number of shared regional anchors is K, and the feature dimension is d. The main computational cost of the anchor-based alignment is approximately proportional to KNd. Since K is much smaller than the number of original image pixels, the alignment module introduces limited additional cost compared with pixel-level registration or dense cross-modal matching. This design improves the scalability of cross-sensor alignment for high-resolution UAV inspection images.

### 2.2. Cross-Sensor Consistency Guided Fusion

After feature alignment between the visible-light images and the infrared thermal images is completed, a cross-sensor consistency guided fusion module is further constructed in this paper. Existing studies on multimodal fusion have shown that the key to fusion strategies lies not only in increasing the number of input information sources but also in determining whether reliable correspondences exist among evidence from different modalities or sensors [32]. Therefore, dual-spectrum evidence is modeled in this paper from three aspects: spatial consistency, semantic consistency, and anomaly consistency. A unified defect representation is then constructed in combination with a reliability-oriented fusion strategy. Before the three consistency measures are introduced, their physical meanings are clarified from the perspective of dual-spectrum blade inspection. In practical visible-infrared inspection, a reliable defect diagnosis should not depend only on the existence of two sensor inputs but on whether the evidence from the two sensors is mutually supportive.

Spatial consistency reflects whether visible structural responses and infrared thermal responses occur in the same or neighboring blade regions. This is important because a true defect often produces coupled morphological and thermal manifestations, whereas background interference or sensor noise usually appears in only one sensor or in unrelated regions. Diagnostic semantic consistency reflects whether the two sensors support similar defect states or abnormal categories. Visible images mainly describe the surface morphology, while infrared images describe the thermal response patterns. When both branches produce similar diagnostic tendencies, the confidence of defect interpretation is increased. Anomaly consistency further evaluates whether the abnormal intensity described by surface structural damage is compatible with the abnormal intensity reflected by thermal responses. This is useful for distinguishing reliable dual-sensor evidence from cases where a weak visual scratch has no thermal implication or where a thermal anomaly is caused by environmental fluctuation rather than structural damage.

Therefore, the three consistency measures correspond to three complementary levels of physical evidence: regional correspondence, diagnostic interpretation, and abnormal severity. By introducing these measures before feature fusion, CG-DSF can suppress isolated, low-quality, or conflicting sensor responses and assign greater importance to evidence that is jointly supported by visible and infrared observations. This provides a more reliable basis for subsequent defect diagnosis and risk grading.

#### 2.2.1. Spatial Consistency Analysis

For offshore wind turbine blade defect diagnosis, if obvious surface texture variations, crack boundaries, or erosion morphology are detected by the visible-light branch in a certain region, while strong thermal responses are also observed by the infrared branch in the same or adjacent region, this region is supported by both the appearance of structural information and by thermal anomaly information and should be assigned greater attention in subsequent fusion. Therefore, spatial consistency analysis is the first step in cross-sensor consistency guided fusion, and its objective is to identify the region-level joint support relationships and potential conflict relationships between dual-spectrum features before fusion.

Let the aligned visible-light feature and infrared thermal feature be denoted as ${\tilde{F}}_{i}^{v}$ and ${\tilde{F}}_{i}^{t}$, respectively, both of which contain K regional tokens. In this paper, the regional attention distributions of the two types of sensors are first obtained through lightweight regional response mapping:

(20) $$A_{i}^{v}=\delta (g_{s}^{v}({\tilde{F}}_{i}^{v})),A_{i}^{t}=\delta (g_{s}^{t}({\tilde{F}}_{i}^{t}))$$

where $A_{i}^{v}$ and $A_{i}^{t}$ denote the response intensities of the visible-light branch and the infrared branch in each region, respectively, and $g_{s}^{v}$ and $g_{s}^{t}$ denote sensor-specific regional response mapping functions. A larger $A_{i,k}^{v}$ indicates greater attention assigned by the visible-light branch to the k-th region, while a larger $A_{i,k}^{t}$ indicates a more significant thermal anomaly response of the infrared thermal image branch in this region.

Infrared thermal anomalies usually exhibit certain diffusion characteristics, and their response regions may not completely coincide with crack boundaries, erosion edges, or peeling contours in visible-light images. Therefore, strict one-to-one positional matching is not adopted in this study. Instead, local neighborhood compensation is performed for the infrared regional response. Let $S_{\rho }$ denote a local smoothing operator with radius ρ. The spatial consistency score is then obtained as:

(21) $$C_{i,k}^{s}=1-\left|A_{i,k}^{v}-S_{\rho }{\left(A_{i}^{t}\right)}_{k}\right|$$

where $C_{i,k}^{s}\in [0,\;1]$ denotes the spatial consistency of the k-th region. When the visible-light response is close to the infrared response after neighborhood compensation, $C_{i,k}^{s}$ is high, indicating strong cross-sensor spatial consistency in this region. When a large difference exists between the two responses, $C_{i,k}^{s}$ is low, indicating a possible sensor response conflict or local interference in this region.

To transfer the spatial consistency result to the subsequent fusion module, a region-level spatial consistency guided weight is further constructed in this paper:

(22) $$G_{i,k}^{s}=C_{i,k}^{s}\cdot \frac{A_{i,k}^{v}+S_{\rho }{\left(A_{i}^{t}\right)}_{k}}{2}$$

This weight considers both the response intensity of the dual sensors in the region and the degree of spatial consistency. When a region exhibits a strong visible-light response, a strong infrared thermal response, and a high spatial consistency simultaneously, the $G_{i,k}^{s}$ increases accordingly, indicating that this region is a key region jointly supported by the dual sensors.

Through this mechanism, regions jointly supported by the dual sensors can be distinguished from potential spatial conflict regions before fusion. Thus, greater attention is assigned to reliable regions in the subsequent fusion process, while inconsistent regions are moderately suppressed.

#### 2.2.2. Semantic Consistency Analysis

After the region-level spatial consistency analysis is completed, it is still necessary to further determine, from the diagnostic semantic level, whether the two types of sensors indicate similar defect types or abnormal states. The semantics here do not refer to textual semantics, but to the defect category tendency, abnormal state judgment, or diagnostic semantic distribution formed by the model based on visible-light structural features and infrared thermal anomaly features, respectively.

In this paper, the two types of features are, respectively, mapped into diagnostic semantic distributions through sensor-specific lightweight semantic prediction heads:

(23) $$p_{i}^{v}=Softmax(h_{sem}^{v}(Pool({\tilde{F}}_{i}^{v})))$$

(24) $$p_{i}^{t}=Softmax(h_{sem}^{t}(Pool({\tilde{F}}_{i}^{t})))$$

where $p_{i}^{v}$ and $p_{i}^{t}$ denote the diagnostic semantic distributions of the visible-light branch and the infrared branch for the current sample, respectively; $h_{sem}^{v}$ and $h_{sem}^{t}$ denote the semantic mapping functions corresponding to the two types of sensors; and Pool denotes the feature aggregation operation. If similar semantic distributions are obtained from the two branches, mutual support is indicated not only at the feature level but also in terms of diagnostic interpretation.

To measure the consistency degree between the two diagnostic semantic distributions, a normalized distribution discrepancy is adopted in this paper to construct the semantic consistency score:

(25) $$C_{i}^{sem}=1-\frac{D_{JS}(p_{i}^{v},p_{i}^{t})}{logC}$$

where $D_{JS}$ denotes the Jensen–Shannon divergence, C denotes the number of diagnostic categories, and $C_{i}^{sem}\in [0,\;1]$. When the judgments of the visible-light branch and the infrared branch on the defect category or abnormal state tend to be consistent, $D_{JS}(p_{i}^{v},p_{i}^{t})$ becomes smaller and $C_{i}^{sem}$ becomes higher. Compared with the direct comparison of final class labels, probability distribution-based semantic consistency can preserve the soft discriminative relationships among different categories and is more suitable for complex inspection samples with weak defects, ambiguous boundaries, and uncertain thermal responses.

By introducing semantic consistency, the model not only focuses on whether the two types of sensors attend to similar regions but also further determines whether similar interpretations of the blade state are formed, thus improving the discriminative reliability of dual-spectrum fusion diagnosis.

#### 2.2.3. Anomaly Consistency Analysis

Semantic consistency can indicate whether the two types of sensors tend to infer similar defect categories or abnormal states, but it cannot fully describe the relationship between anomaly intensities. For example, a certain region may appear as only a slight surface scratch in the visible-light image, whereas a strong thermal anomaly may be observed in the infrared thermal image. This situation may indicate that potential internal damage is more severe than the surface damage. Therefore, anomaly consistency analysis is not focused on determining whether the defect categories are identical but on comparing whether the anomaly degrees characterized by the two types of sensors are mutually supportive.

In this paper, the visual anomaly intensity and thermal anomaly intensity are obtained through sensor-specific anomaly scoring functions:

(26) $$r_{i}^{v}=\sigma (h_{a}^{v}(Pool({\tilde{F}}_{i}^{v}))),r_{i}^{t}=\sigma (h_{a}^{t}(Pool({\tilde{F}}_{i}^{t})))$$

where $r_{i}^{v}\in [0,\;1]$ denotes the structural anomaly degree estimated by the visible-light branch, $r_{i}^{t}\in [0,\;1]$ denotes the thermal anomaly degree estimated by the infrared branch, and $h_{a}^{v}$ and $h_{a}^{t}$ denote the anomaly scoring functions of the visible-light branch and the infrared branch, respectively. The visible-light anomaly intensity is mainly derived from structural changes such as crack boundaries, surface texture damage, erosion regions, and coating peeling, whereas the infrared anomaly intensity is mainly derived from thermal manifestations such as relative thermal anomalies, temperature gradient variations, local hot spots, and thermal response discontinuity.

After the two types of anomaly intensities are obtained, their matching degree is defined in this paper as the anomaly consistency score:

(27) $$C_{i}^{a}=1-\left|r_{i}^{v}-r_{i}^{t}\right|$$

where $C_{i}^{a}\in [0,\;1]$. When the visual anomaly intensity is close to the infrared thermal anomaly intensity, $C_{i}^{a}$ becomes higher, indicating a good match between the structural damage evidence and the thermal anomaly evidence in terms of anomaly degree.

Through anomaly consistency analysis, abnormal samples jointly confirmed by dual sensors can be distinguished from suspicious samples dominated by a single sensor. As a result, the final unified representation contains both structural damage evidence and thermal anomaly evidence, while diagnostic information carried by anomaly intensity differences is also preserved.

#### 2.2.4. Reliability-Aware Fusion Strategy

The dual-sensor information has been aligned, and the consistency analysis has been completed in the preceding sections. The cooperative relationship between the visible-light branch and the infrared thermal image branch has been characterized from three perspectives: regional correspondence, diagnostic tendency, and anomaly intensity. However, these consistency results cannot be directly regarded as the final fusion result. In practical offshore wind turbine blade inspection, visible-light images and infrared thermal images are affected by multiple factors. Therefore, fixed fusion weights should not be adopted. Instead, both sensor data quality and cross-sensor consistency relationships should be considered. Based on this idea, a reliability-oriented fusion strategy is constructed in this paper, so that the contributions of visible-light features and infrared thermal features during fusion can be adaptively adjusted according to the observation conditions of the different samples and the dual-sensor response relationships.

First, the aforementioned three types of consistency results are integrated to form a sample-level cross-sensor consistency description. Spatial consistency reflects whether the dual sensors focus on similar blade regions, semantic consistency reflects whether similar diagnostic tendencies are formed by the two sensors, and anomaly consistency reflects whether the anomaly degrees characterized by the two sensors are matched. To avoid the fusion judgment being dominated by a single consistency indicator, a learnable mapping is adopted in this paper for joint modeling of the three types of consistency information:

(28) $$c_{i}=\sigma \left(\omega_{c}^{T}\left[C_{i}^{s},C_{i}^{sem},C_{i}^{a}\right]+b_{c}\right)$$

where $C_{i}^{s}$, $C_{i}^{sem}$, and $C_{i}^{a}$ denote the spatial consistency, semantic consistency, and anomaly consistency of the i-th sample, respectively; $w_{c}$ and $b_{c}$ are learnable parameters; and $c_{i}\in [0,\;1]$ denotes the comprehensive consistency score. A higher $c_{i}$ indicates stronger mutual support between visible-light structural information and infrared thermal response information across multiple levels and, thus, a higher confidence level for dual-spectrum fusion.

On the basis of comprehensive consistency modeling, the intrinsic reliability of different sensors in the current sample is further estimated in this paper. For the visible-light branch, reliability is mainly associated with image clarity, defect region visibility, local texture distinguishability, and the degree of background interference. For the infrared thermal image branch, reliability is mainly associated with thermal contrast, thermal anomaly continuity, noise level, and the degree of background thermal drift. Since these factors directly affect the credibility of the features from the corresponding branches, sensor-specific reliability estimation functions are adopted in this paper to obtain visible-light reliability and infrared reliability:

(29) $$q_{i}^{v}=\sigma (h_{q}^{v}(Pool({\tilde{F}}_{i}^{v}))),q_{i}^{t}=\sigma (h_{q}^{t}(Pool({\tilde{F}}_{i}^{t})))$$

where $q_{i}^{v}$ and $q_{i}^{t}$ denote the sample-level reliability scores of the visible-light branch and the infrared thermal image branch, respectively; $h_{q}^{v}$ and $h_{q}^{t}$ denote the reliability estimation functions corresponding to the two types of sensors; and Pool denotes the feature aggregation operation. With this design, the importance of different sensor features can be dynamically adjusted according to the actual imaging quality of the current input sample, rather than assuming that visible-light information and infrared information are equally reliable across all samples.

Subsequently, the intrinsic sensor reliability and the cross-sensor comprehensive consistency are jointly used for fusion weight generation. Specifically, the reliability weights of the visible-light branch and the infrared branch are defined as:

(30) $$\left[\alpha_{i}^{v},\alpha_{i}^{t}\right]=Softmax\left(\left[q_{i}^{v}\cdot c_{i},q_{i}^{t}\cdot c_{i}\right]\right)$$

where $\alpha_{i}^{v}$ and $\alpha_{i}^{t}$ denote the fusion weights of the visible-light feature and the infrared thermal feature in the current sample, respectively, and satisfy $\alpha_{i}^{v}+\alpha_{i}^{t}=1$. When a sensor exhibits higher data quality in the current sample and stronger overall consistency exists between the dual sensors, a more reasonable contribution is assigned to the corresponding feature during fusion.

However, reliability-aware weighting does not imply that the sensor with a high weight is simply retained while the sensor with a low weight is discarded. For offshore wind turbine blade defect diagnosis, weak or inconsistent responses from a single sensor may still contain valuable information. Therefore, a soft weighting strategy is adopted in this paper to perform reliability correction on the two types of features, rather than hard filtering. The dual sensor features, after reliability adjustment, can be expressed as:

(31) $$F_{i}^{v,r}=\alpha_{i}^{v}{\tilde{F}}_{i}^{v},F_{i}^{t,r}=\alpha_{i}^{t}{\tilde{F}}_{i}^{t}$$

where $F_{i}^{v,r}$ and $F_{i}^{t,r}$ denote the reliability-weighted visible-light feature and infrared thermal feature, respectively. Through this process, complementary dual-spectrum information is retained, while the interference of low-quality or conflicting features in the subsequent construction of the unified representation is reduced.

In implementation, the sensor reliability scores are generated by lightweight prediction heads attached to the visible-light and infrared branches. The two reliability scores are normalized through a softmax function to obtain sample-adaptive modality weights. These weights are learned jointly with the whole diagnostic network under the supervision of defect localization, defect classification, and risk grading objectives. Therefore, the reliability-aware fusion module does not rely on manually assigned sensor weights. Instead, it learns to adjust the contribution of visible-light and infrared features according to sample-level imaging quality and cross-sensor consistency.

Overall, the reliability-oriented fusion strategy associates intrinsic sensor quality with cross-sensor consistency, so that the fusion process is transformed from fixed weighting or simple concatenation into sample adaptive reliability regulation. More stable weighted feature inputs are, therefore, provided for the subsequent construction of a consistency-guided unified representation.

#### 2.2.5. Consistency-Guided Unified Representation

The construction of the consistency-guided unified representation is the final output stage of the cross-sensor consistency-guided fusion module. Its objective is to integrate visible-light structural information, infrared thermal anomaly information, and the aforementioned consistency analysis results into a unified defect representation on the basis of reliability weighting. In constructing the unified representation, fusion information, consistency information, and discrepancy information are simultaneously retained, so that the final representation possesses both comprehensiveness and diagnostic discriminability.

Specifically, the spatial consistency guidance map $G_{i}^{s}$ is first used to regionally modulate the dual-spectrum features, so that greater attention is assigned to the blade regions jointly supported by visible-light images and infrared thermal images in the spatial domain. This process can be expressed as:

(32) $$H_{i}^{v}=G_{i}^{s}⊙F_{i}^{v,r},H_{i}^{t}=G_{i}^{s}⊙F_{i}^{t,r}$$

where $H_{i}^{v}$ and $H_{i}^{t}$ denote the visible-light feature and infrared thermal feature after spatial consistency modulation, respectively, and ⊙ denotes element-wise multiplication. Through this operation, regions with consistent spatial responses are enhanced in the feature representation, while regions with spatial conflicts or strong background interference are moderately suppressed.

On this basis, a cross-sensor discrepancy representation is further constructed to preserve information that is not fully consistent between the visible-light images and the infrared thermal images but may still be diagnostically meaningful:

(33) $$D_{i}=\left|F_{i}^{v,r}-F_{i}^{t,r}\right|$$

where $D_{i}$ denotes the discrepancy response between the dual-spectrum features. This discrepancy term is not intended to amplify the sensor conflicts, but to provide additional discriminative cues for the model.

Subsequently, the spatially modulated visible-light feature, the spatially modulated infrared feature, the dual-spectrum discrepancy feature, and the three types of consistency scores are jointly input into the unified representation construction function:

(34) $$U_{i}=H\left(\left[H_{i}^{v},H_{i}^{t},D_{i},C_{i}^{s},C_{i}^{sem},C_{i}^{a}\right]\right)$$

where H denotes the unified representation mapping function, which can be composed of a linear projection and a lightweight nonlinear transformation. $U_{i}$ is jointly regulated by consistency relationships and reliability weights, and denotes the final consistency-guided unified representation. This representation contains not only the surface structural damage information provided by the visible-light branch and the thermal anomaly response information provided by the infrared branch, but also spatial consistency, semantic consistency, and anomaly consistency, which are explicitly introduced as fusion constraints. Accordingly, the support degree of dual-sensor evidence can be comprehensively evaluated in a unified feature space.

**Figure 4:**
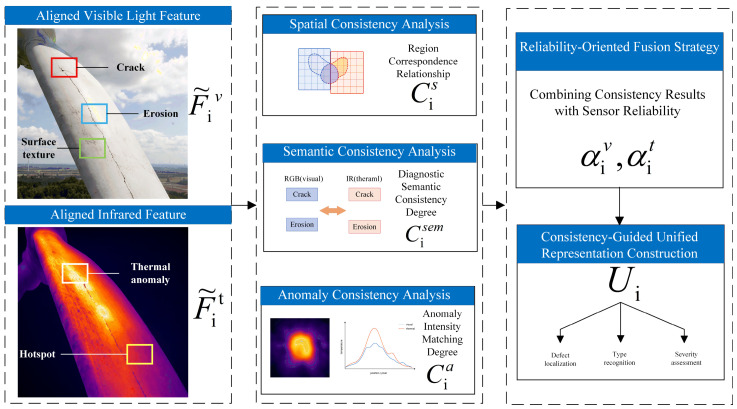
Cross-sensor consistency-guided fusion process.

The cross-sensor consistency-guided fusion module is shown in Figure 4:

### 2.3. Defect Diagnosis and Severity Assessment

After the consistency-guided unified representation $U_{i}$ is obtained, a defect localization and type identification module is further constructed in this paper, through which the fused dual-spectrum features are transformed into interpretable diagnostic results for blade defects.

#### 2.3.1. Defect Localization and Classification

Previous studies on intelligent wind turbine blade inspection usually formulate defect detection as defect region localization, defect mask extraction, or defect category identification, with the localization or segmentation results further used to support the defect size quantification and damage assessment. Therefore, this stage is not simply modeled as an image-level classification task in this paper. Instead, it is defined as a joint diagnostic process for blade defect regions, in which the defect candidate regions and their corresponding defect types or abnormal states are output based on the unified representation $U_{i}$.

Specifically, given the consistency-guided unified representation $U_{i}$ of the i-th sample, the output of the defect diagnosis module is expressed as:

(35) $$O_{i}={\left\{\left({\hat{B}}_{i,n},{\hat{y}}_{i,n},{\hat{p}}_{i,n}\right)\right\}}_{n=1}^{N_{i}}$$

where ${\hat{B}}_{i,n}$ denotes the n-th defect candidate region, ${\hat{y}}_{i,n}$ denotes the corresponding defect type or abnormal state, ${\hat{p}}_{i,n}$ denotes the confidence of the diagnostic result, and $N_{i}$ denotes the number of candidate defects predicted in the current sample.

During defect localization, potential defect regions on the blade surface are generated according to the regional responses in $U_{i}$. When defect boundaries are weak, surface textures are similar to the background or infrared thermal anomalies exhibit local diffusion, the stability of candidate region extraction can be improved by the complementary relationship between the dual-spectrum information in the unified representation. During type identification, category discrimination is further performed for each candidate region to obtain the corresponding defect type or abnormal state. For candidate regions with more evident visible-light evidence, structural information such as crack contours, surface peeling, leading edge erosion, and texture damage is mainly used for discrimination. For candidate regions with more evident infrared evidence, thermal information such as local hot spots, temperature gradient variations, and thermal response continuity is incorporated to support the judgment.

#### 2.3.2. Severity Evaluation Mechanism

After defect candidate region localization and type identification are completed, a severity assessment mechanism is further constructed in this paper. The severity assessment is established based on the defect candidate region ${\hat{B}}_{i,n}$, defect type ${\hat{y}}_{i,n}$, and diagnostic confidence ${\hat{p}}_{i,n}$ output in the previous section, rather than simply equating the classification probability with the defect severity.

For the n-th defect candidate region in the i-th sample, severity is mainly determined by three types of evidence. The first type is structural damage evidence, which mainly reflects the defect area, boundary damage, texture anomaly, and morphological extension in visible-light images, and can be used to describe the spatial impact of surface damage, such as cracks, leading edge erosion, and coating peeling. The second type is thermal anomaly evidence, which mainly reflects the relative thermal anomaly intensity, temperature gradient variation, and local hot spot distribution in infrared thermal images, and can complement the thermal response information that is difficult to directly characterize through visible-light images. The third type is consistency evidence, which is derived from spatial consistency, semantic consistency, and anomaly consistency analysis, and is used to measure whether the visible-light structural response and infrared thermal response are mutually supportive. If a candidate region exhibits evident structural damage, stable thermal anomaly, and high cross-sensor consistency simultaneously, its severity assessment result is more reliable.

Based on the above considerations, the severity of a candidate defect region is modeled in this paper as an integrated scoring process based on multisource diagnostic evidence:

(36) $$S_{i,n}=\sigma (h_{s}\left(\left[z_{i,n}^{str},z_{i,n}^{th},z_{i}^{con},e\left({\hat{y}}_{i,n}\right),{\hat{p}}_{i,n}\right]\right))$$

where $S_{i,n}\in [0,\;1]$ denotes the severity score of the n-th candidate defect, $z_{i,n}^{str}$ denotes the structural damage evidence, $z_{i,n}^{th}$ denotes the thermal anomaly evidence, $z_{i}^{con}$ denotes the consistency evidence, $e({\hat{y}}_{i,n})$ denotes the defect type embedding or class impact factor, and $h_{s}$ denotes the severity mapping function.

When multiple candidate defect regions exist in the same sample, region-level severity aggregation is adopted in this paper to obtain the sample-level severity:

(37) $$S_{i}=maxS_{i,n},1\leq n\leq N_{i}$$

where $S_{i}$ denotes the overall severity score of the i-th sample. Maximum aggregation is adopted to highlight the influence of the most severe defect on the blade state, which is suitable for risk screening requirements in inspection scenarios.

#### 2.3.3. Risk-Level Assessment

After the sample level severity score $S_{i}$ is obtained, a risk-level assessment mechanism is further constructed in this study, with the objective of providing engineering-interpretable risk grading results according to defect severity, the support degree of cross-sensor evidence, and diagnostic confidence.

Specifically, the risk levels are defined as three ordinal categories: low risk, medium risk, and high risk. The grading criteria are established according to four types of information: visible structural evidence, infrared thermal evidence, cross-sensor consistency evidence, and inspection-priority requirements. Visible structural evidence mainly reflects crack morphology, leading-edge erosion, coating damage, defect area, and boundary extension. Infrared thermal evidence mainly reflects local hot spots, relative thermal anomaly intensity, thermal gradient discontinuity, and suspected subsurface abnormal responses. Cross-sensor consistency evidence reflects whether visible and infrared observations jointly support the same defect-related region, diagnostic tendency, and abnormal degree. The inspection-priority requirement is used to connect the model output with practical review and maintenance decisions. To avoid risk levels being determined solely by a single severity score or classification confidence, a comprehensive risk score is introduced in this paper. This score reflects not only the severity of the defect itself but also the diagnostic confidence and the support degree of dual-spectrum sensing evidence:

(38) $$R_{i}^{score}=h_{r}\left(S_{i},C_{i}^{s},C_{i}^{sem},C_{i}^{a},{\bar{p}}_{i}\right)$$

where $R_{i}^{score}$ denotes the comprehensive risk score of the i-th sample, $S_{i}$ denotes the sample-level severity score, ${\bar{p}}_{i}$ denotes the comprehensive diagnostic confidence of the candidate defect region. In this study, $R_{i}^{score}$ is normalized to the interval [0, 1]. A larger value indicates a stronger defect severity, a higher diagnostic confidence, and a stronger cross-sensor support.

After the comprehensive risk score is obtained, two thresholds, $\tau_{1}$ and $\tau_{2}$, are used to map the continuous risk score into ordinal risk levels:

(39) $$L_{i}=\left\{\begin{array}{c} Low,{\;R}_{i}^{score}<\tau_{1}\\ Medium,{\tau_{1}\leq R}_{i}^{score}<\tau_{2}\\ High,\;R_{i}^{score}\geq \tau_{2} \end{array}\right.$$

where $\tau_{1}$ and $\tau_{2}$ denote the risk grading thresholds between low and medium risk and between medium and high risk, respectively. In the experiments, the thresholds were determined using only the validation set. Specifically, the distribution of risk scores on the validation set was first analyzed together with the expert-reviewed ordinal labels. The thresholds were then selected to maintain the ordinal consistency between the predicted risk levels and the reference risk labels, while also considering the inspection-priority requirement that high-risk samples should be concentrated in the priority review set. Once determined, $\tau_{1}$ and $\tau_{2}$ were fixed and directly applied to the test set. No test-set information was used for threshold tuning.

The purpose of the risk-level assessment is to further transform the model output from simple defect detection results into graded diagnostic information for operation and maintenance scenarios. Through this mechanism, not only can defect location and type be provided by the model, but also the severity and risk level, so that inspection priorities and maintenance sequences can be arranged according to different risk levels.

## 3. Experiments and Results

### 3.1. Dataset Construction and Annotation Protocol

The experimental data were derived from the public AQUADA-GO project, which provides optical and infrared video data of in-service wind turbine blades. In this study, paired visible and infrared samples were constructed at the frame level. Each sample consisted of one visible-light frame and its corresponding infrared thermal frame acquired from the same inspection sequence. Frames with severe blade-region loss, obvious motion blur, excessive background occupation, or invalid cross-sensor correspondence were removed. The pairing relationship between visible-light and infrared frames was strictly preserved during sample construction, data partitioning, training, and testing.

To improve data transparency, the dataset statistics and risk-label distribution are summarized in Table 1. The dataset was divided into training, validation, and test sets according to paired inspection sequences rather than random frame-level splitting. This strategy prevents frames from the same visible–infrared sequence from appearing in different subsets and reduces the risk of data leakage. The training set was used for model parameter learning. The validation set was used for hyperparameter selection, early stopping, and risk-threshold determination, and the test set was used only for final performance evaluation.

To support defect diagnosis and severity-oriented risk grading, an annotation protocol was established before model training. The annotation process was performed on the original paired samples rather than on model-generated feature maps, attention maps, consistency scores, or predicted risk scores. For each sample, the visible-light image was reviewed to identify surface morphological defects, such as cracks, leading-edge erosion, coating damage, and other surface abnormalities. The corresponding infrared thermal image was reviewed to identify local thermal anomalies, abnormal temperature gradients, and suspected thermal discontinuity regions. Based on the joint visible–infrared review, each sample was assigned a defect category and a risk level.

The risk level was divided into low, medium, and high risk according to visible structural evidence, infrared thermal evidence, and cross-sensor support. Low-risk samples corresponded to normal or weak abnormal responses that required only routine inspection tracking. Medium-risk samples corresponded to visible surface damage or thermal anomaly responses requiring focused review in subsequent inspections. High-risk samples corresponded to obvious structural damage, strong thermal anomaly responses, or abnormal regions requiring priority manual review and further testing. The ground-truth labels were generated before model training and remained fixed during all experiments.

To reduce the subjectivity of risk-label construction, a consensus-based annotation procedure was adopted. Two annotators independently reviewed the paired visible–infrared samples according to the predefined grading criteria, including visible structural damage, infrared thermal anomaly response, and cross-sensor evidence support. For samples with inconsistent annotations, an expert review was further conducted, and the final risk label was determined through discussion and consensus. Since the low-, medium-, and high-risk labels are ordinal, weighted Cohen’s kappa was used to evaluate the inter-annotator agreement before consensus correction. The obtained weighted kappa value was 0.84, indicating substantial agreement between the annotators. All risk labels were finalized before model training and were not derived from model-generated attention maps, consistency scores, feature representations, or predicted risk scores. The predefined grading criteria used in annotation were consistent with the risk-level definitions in Section 2.3.3, and the thresholds for model output were selected on the validation set after the annotation labels were finalized.

### 3.2. Experimental Protocol and Evaluation Settings

In this paper, the training, validation, and test sets are divided using paired visible-light and infrared thermal video sequences as the basic units. All samples within the same paired sequence are assigned to the same subset, and the one-to-one correspondence between visible-light images and infrared thermal images is consistently preserved. The training set is used for model parameter learning. The validation set is used for hyperparameter selection, model early stopping, and risk grading threshold determination, and the test set is used only for final performance evaluation. In terms of input processing, both visible-light images and infrared thermal images are resized to a unified resolution and standardized according to their respective sensor characteristics. Pixel normalization and blade region enhancement are mainly performed on visible-light images, while temperature response normalization and background thermal response correction are mainly performed on infrared thermal images.

Evaluation metrics are set according to the diagnostic tasks. First, IoU and region hit rate are adopted for defect localization to evaluate the overlap between the predicted regions and the reference annotations. Accuracy and Macro F1 are adopted for defect type identification to evaluate the classification performance. AUROC, AUPRC, and confusion matrix analysis are adopted for severity and risk-level assessment to evaluate the risk discrimination capability and probability reliability. Considering that low-, medium-, and high-risk levels are ordinal rather than purely nominal categories, two additional ordinal evaluation metrics were introduced in the revised manuscript. The three risk levels were encoded as zero, one, and two, respectively. Weighted Cohen’s kappa was used to evaluate the agreement between the predicted and reference risk levels while considering the distance between ordinal categories. Ordinal MAE was used to measure the average absolute deviation between the predicted and reference risk levels. These two metrics complement Accuracy, Macro F1, AUROC, and AUPRC by evaluating whether the predicted results preserve the ordinal relationship among different risk levels.

In addition to the classification and ordinal grading metrics, engineering-oriented inspection-priority metrics were further adopted to evaluate the practical usefulness of the risk scores. Specifically, Precision@k was used to measure the proportion of true high-risk samples among the top k% samples ranked by the predicted risk score. In this study, Precision@k can also be interpreted as the high-risk hit rate under limited inspection resources. Lift@k was used to compare the high-risk hit rate of the model-guided review strategy with that of random screening. These metrics evaluate whether the proposed method can concentrate high-risk samples in the priority review set, which is directly related to maintenance decision reliability.

For model training, all deep learning-based models were trained using the same training and validation partitions. The AdamW optimizer was adopted, with an initial learning rate of 1 × 10^−4^, a weight decay of 1 × 10^−4^, and a batch size of 16. The maximum number of training epochs was set to 100, and early stopping was applied when the validation loss did not decrease for 15 consecutive epochs. Data augmentation, including random cropping, slight rotation, brightness perturbation, thermal contrast perturbation, and local noise injection, was used only on the training set.

The training objective of CG-DSF consists of defect localization loss, defect classification loss, risk grading loss, and consistency regularization loss. The localization loss constrains the predicted defect region. The classification loss supervises the defect category prediction, and the risk grading loss supervises the low-, medium-, and high-risk outputs. The consistency regularization term encourages mutually supportive visible-light and infrared evidence while suppressing conflicting sensor responses.

All models are compared under the same data partition, input size, and training strategy, so that the experimental results mainly reflect the cross-sensor fusion and risk discrimination capabilities of the models themselves.

### 3.3. Overall Diagnostic Performance

To verify the effectiveness of the proposed CG-DSF method in the risk-level diagnosis task, five representative models were selected for comparison, including XGBoost2.0.3, ViT, YOLOv8 framework 8.1.0, a multimodal Transformer, and CG-DSF. XGBoost was retained as a strong traditional machine learning baseline based on gradient boosting trees. ViT was selected as a representative vision Transformer model to evaluate the performance of modern image-based deep learning in blade defect diagnosis. YOLOv8 was introduced as a representative object detection model for defect localization and category recognition. The multimodal Transformer was selected as a representative deep visible–infrared fusion baseline to evaluate the performance of attention-based multimodal feature interaction. The proposed CG-DSF was compared with these models to examine whether explicit cross-sensor consistency modeling can provide additional advantages for defect diagnosis and risk grading.

All compared models were trained and evaluated under the same data partition, input preprocessing protocol, and evaluation metrics. For XGBoost, organized diagnostic features were used as inputs for three-level risk classification. For ViT, the final prediction head was adapted to the risk grading task. For YOLOv8, defect localization and category prediction results were first obtained, and the region-level diagnostic outputs were then mapped to sample-level risk grades according to the same risk evaluation protocol. For the multimodal Transformer and CG-DSF, paired visible and infrared images were used as dual-spectrum inputs. This experimental design allows the comparison to evaluate the overall diagnostic performance of CG-DSF against a traditional machine learning baseline, modern image-based deep learning models, an object detection model, and a deep multimodal fusion model.

#### 3.3.1. External Model Comparison

**Figure 5:**
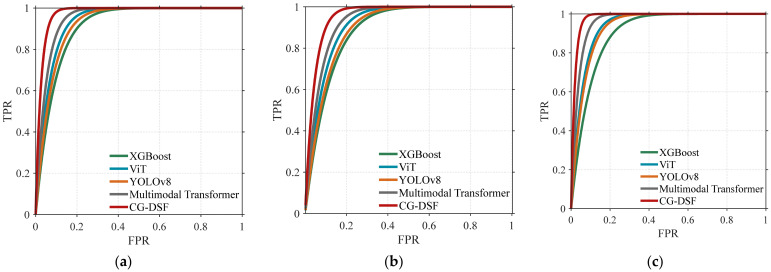
ROC curve comparison: (**a**) low risk, (**b**) medium risk, (**c**) high risk.

As shown in Table 2 and Figure 5, CG-DSF achieves the highest AUROC values among the five compared models for low-risk, medium-risk, and high-risk samples, reaching 0.974, 0.956, and 0.981, respectively. Compared with XGBoost, ViT, YOLOv8, and the multimodal Transformer, CG-DSF maintains a higher true-positive rate and a lower false-positive rate under different discrimination thresholds. This indicates that the proposed method provides stronger risk discrimination capability across all three risk levels.

The improvement is particularly evident for the high-risk category. CG-DSF obtains an AUROC of 0.981, which is higher than XGBoost, ViT, YOLOv8, and the multimodal Transformer. This result suggests that cross-sensor consistency modeling is effective for distinguishing high-risk blade anomaly samples from low- and medium-risk samples. Since high-risk samples are the primary concern in offshore wind turbine blade inspection, the improved AUROC performance demonstrates the potential of CG-DSF for priority risk screening and targeted inspection review.

**Figure 6:**
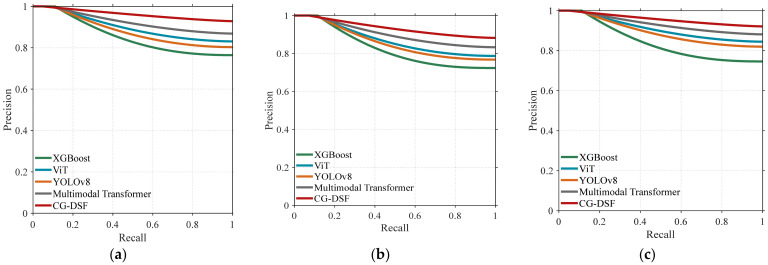
PR curve comparison: (**a**) low risk, (**b**) medium risk, (**c**) high risk.

As shown in Table 3 and Figure 6, CG-DSF also achieves the best AUPRC performance for the low-risk, medium-risk, and high-risk categories, with values of 0.962, 0.935, and 0.958, respectively. These values are higher than those of XGBoost, ViT, YOLOv8, and the multimodal Transformer, indicating that the proposed method maintains better precision and recall in risk sample identification.

Compared with AUROC, AUPRC is more sensitive to class imbalance and is, therefore, more suitable for evaluating the identification capability of risk samples when the proportions of different risk levels are uneven. The superior AUPRC results show that CG-DSF not only improves the overall discrimination performance but also reduces missed and false identification of risk samples. This advantage is important for offshore blade inspection because a reliable risk screening model should concentrate high-risk samples in the priority review set while avoiding excessive false alarms.

As shown in Table 4, CG-DSF achieves the highest accuracy and Macro F1 among all five compared models, reaching 0.924 and 0.923, respectively. Compared with XGBoost, YOLOv8, ViT, and the multimodal Transformer, CG-DSF shows a stronger overall classification correctness and a more balanced recognition performance across different risk levels.

Since low-, medium-, and high-risk levels are ordinal, Table 4 further reports the weighted Kappa and ordinal MAE. CG-DSF achieves the highest weighted Kappa of 0.908 and the lowest ordinal MAE of 0.080. This indicates that the predicted risk levels of CG-DSF are more consistent with the reference ordinal labels and that the average grading deviation is smaller than that of the compared models. These results demonstrate that CG-DSF not only improves nominal classification performance but also better preserves the ordinal relationship among different risk levels.

**Figure 7:**
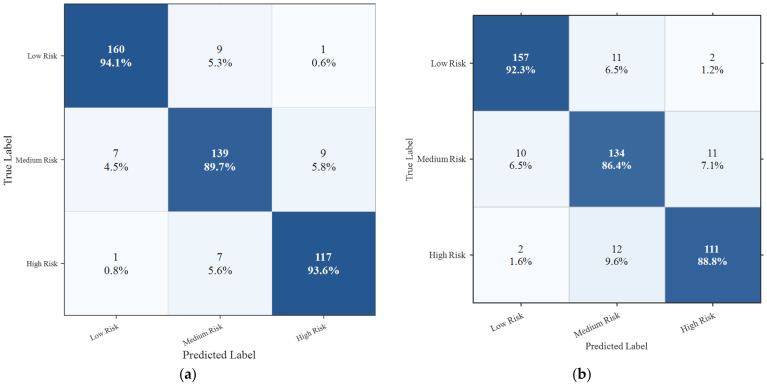

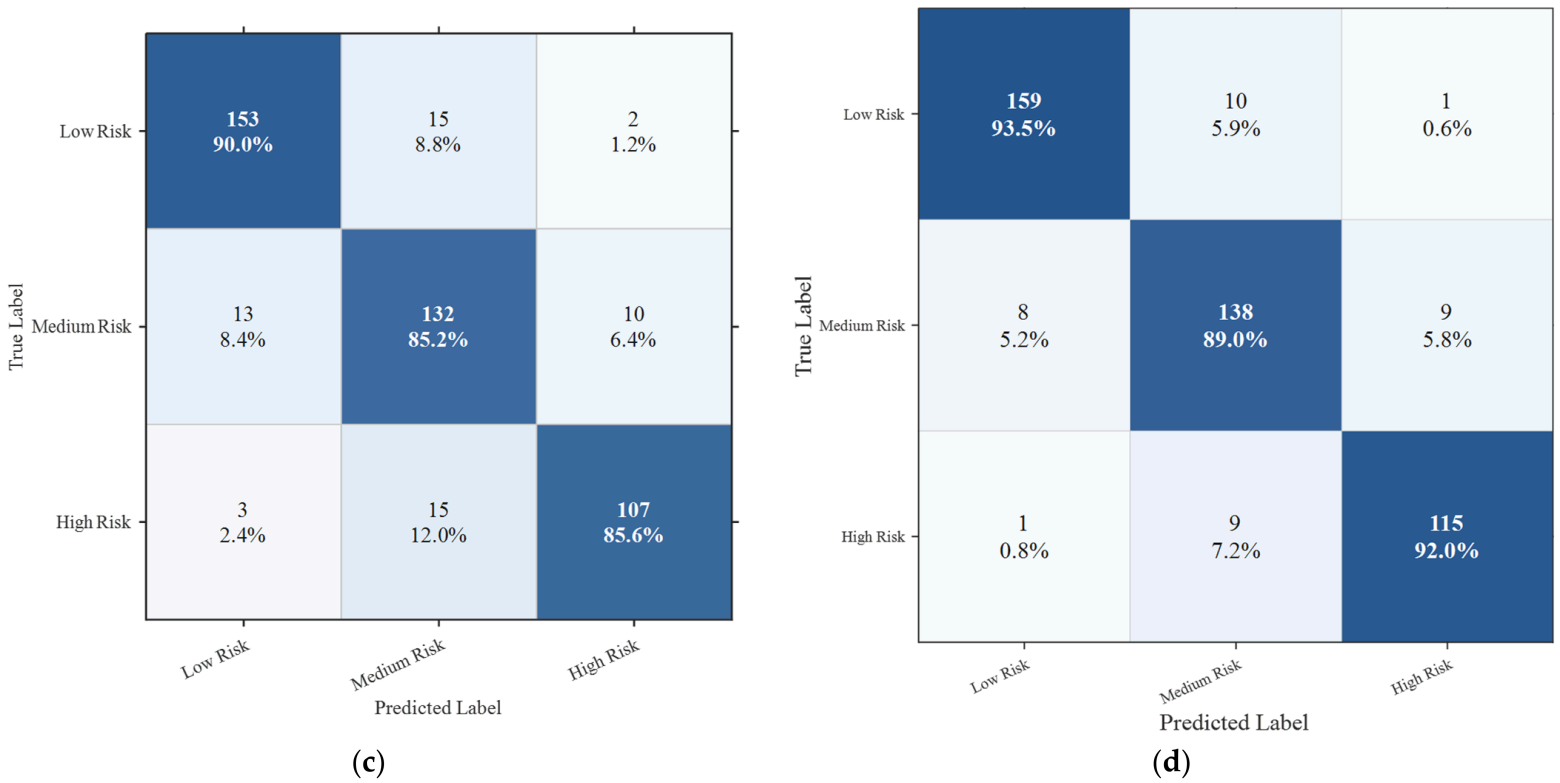
Confusion matrices comparison: (**a**) CG-DSF, (**b**) ViT, (**c**) XGBoost, (**d**) multimodal Transformer.

Figure 7 presents the confusion matrices of four representative models, including XGBoost, ViT, the multimodal Transformer, and CG-DSF. Compared with the representative baselines, CG-DSF produces fewer misclassifications among adjacent risk levels and shows more stable recognition for high-risk samples. Most errors of the comparison models occur between medium-risk and high-risk samples, indicating that distinguishing severe but visually or thermally ambiguous defects remains challenging. By jointly considering the visible structural evidence, infrared thermal anomaly evidence, and cross-sensor consistency information, CG-DSF reduces such confusion and improves the stability of risk-level discrimination.

#### 3.3.2. Category-Wise Diagnostic Performance Analysis

To further analyze the diagnostic stability of CG-DSF across different defect categories, Precision, Recall, and F1 score are calculated for each type of defect sample in this section. The diagnostic results of CG-DSF for the different defect categories are presented in Table 5.

As shown in Table 5, CG-DSF achieves stable recognition performance for cracks, leading-edge erosion, coating damage, and thermal anomaly or delamination anomaly, with the F1 score of each category maintained at a high level. Among them, the crack category obtains the highest F1 score, indicating that strong representation capability is provided by the visible-light branch for crack boundaries, local texture variations, and slender structural damage. Favorable results are also obtained for the thermal anomaly or delamination anomaly category, demonstrating that effective complementary information can be provided by the infrared thermal image branch for abnormal regions with inconspicuous visual appearance.

### 3.4. Ablation Experiments

To further analyze the contribution of each component in CG-DSF, the ablation experiments were expanded from both modality-level and module-level perspectives. The modality level ablation was designed to examine whether visible-light and infrared thermal data provide complementary diagnostic evidence under the same backbone setting and diagnostic head. Specifically, CG-DSF-RGB denotes the variant that retains only the visible-light branch and uses the same diagnostic head as the complete model, while CG-DSF-IR denotes the variant that retains only the infrared thermal branch and uses the same diagnostic head. Direct fusion denotes the variant that directly concatenates visible and infrared features without cross-sensor consistency modeling. These variants were used to verify whether the performance gain of CG-DSF comes from dual-spectrum complementary sensing rather than only from a larger model structure.

**Figure 8:**
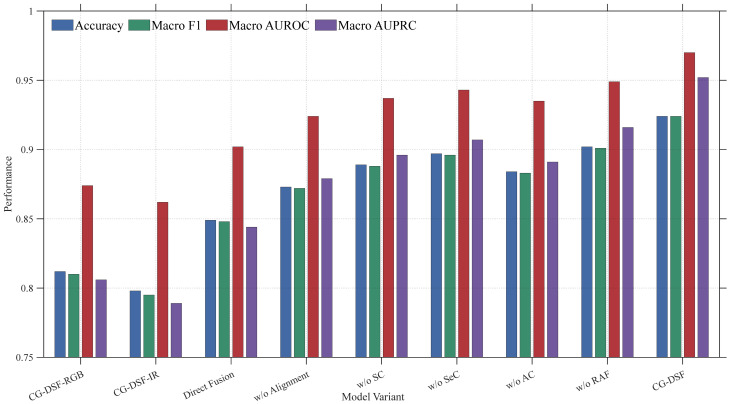
Visualization of ablation performance for different CG-DSF variants.

The module-level ablation was designed to verify the effectiveness of the key components in the proposed framework. The variant without alignment removes the cross-sensor feature alignment module. The variants without spatial consistency analysis (*w*/*o* SC), without semantic consistency analysis (*w*/*o* SeC), and without anomaly consistency analysis (*w*/*o* AC) remove spatial consistency analysis, diagnostic semantic consistency analysis, and anomaly consistency analysis, respectively. The variant without the reliability-aware fusion strategy (*w*/*o* RAF) removes the reliability-aware fusion strategy and adopts ordinary weighted fusion instead. All ablation variants were trained and evaluated under the same data partition, input size, training strategy, and evaluation metrics. Therefore, the performance differences mainly reflect the contribution of the corresponding modality or module, rather than the differences in data splitting or training settings. Table 6 presents the diagnostic results of different ablation models:

As shown in Table 6 and Figure 8, the complete CG-DSF achieves the best performance among all variants, with accuracy, macro F1, macro AUROC, and macro AUPRC reaching 0.924, 0.924, 0.970, and 0.952, respectively. This result indicates that the proposed framework benefits from the joint use of dual-spectrum inputs, cross-sensor alignment, consistency modeling, and reliability-aware fusion.

The modality level results show that CG-DSF-RGB and CG-DSF-IR are both inferior to the complete model. This confirms that visible-light images and infrared thermal images provide complementary diagnostic evidence. Direct fusion performs better than the single-modality variants, but remains clearly lower than CG-DSF, suggesting that simple feature concatenation is insufficient for reliable visible infrared fusion.

The module-level results further show that removing cross-sensor alignment, spatial consistency, semantic consistency, anomaly consistency, or reliability-aware fusion leads to performance degradation. Among these variants, *w*/*o* AC and *w*/*o* alignment show relatively obvious decreases, indicating that anomaly consistency and feature-level alignment are important for stable risk grading. Overall, the ablation results demonstrate that each component contributes to the final performance, and the complete CG-DSF provides the most reliable risk-level diagnosis.

### 3.5. Risk-Score Validation and Inspection-Priority Evaluation

After the analysis of overall diagnostic performance and ablation experiments, the grading consistency of the risk scores output by CG-DSF and their application value in the inspection review scenarios are further investigated in this paper. For this purpose, the distributions of risk scores for samples with different risk levels in the test set are summarized, and the results are shown in Table 7.

As shown in Table 7, a favorable monotonic correspondence is observed between the risk scores output by CG-DSF and the sample risk levels. This indicates that the proposed method can provide not only discrete risk levels but also discriminative continuous risk scores. Meanwhile, the standard deviation results show that certain fluctuations still exist within each risk level, which is associated with variations in actual blade defect manifestations.

To further evaluate the engineering usefulness of CG-DSF under limited inspection resources, an inspection-priority analysis was conducted. In practical offshore blade inspection, maintenance engineers usually cannot manually review all samples immediately. Therefore, a reliable risk model should rank truly high-risk samples as early as possible. Based on the continuous risk scores output by CG-DSF, the samples were sorted in descending order, and the high-risk hit rate and Lift@k were calculated for the top-ranked review sets.

As shown in Table 8, CG-DSF shows favorable inspection-priority performance under limited review resources. When only the top 10% of samples with the highest predicted risk scores are reviewed, the high-risk hit rate reaches 0.846, and the corresponding Lift@k is 3.05. This means that the model-guided review strategy identifies high-risk samples at more than three times the rate of random screening. When the review range is expanded to the top 20% and top 30%, the high-risk hit rate decreases to 0.742 and 0.631, respectively, but remains clearly higher than the random baseline. The corresponding Lift@k values are 2.67 and 2.27, indicating that high-risk samples are still effectively concentrated in the priority review queue. These results demonstrate that the risk scores produced by CG-DSF are not only discriminative in classification metrics but also useful for practical inspection scheduling and maintenance decision support.

### 3.6. Robustness and Cross-Sensor Misalignment Analysis

To further evaluate the robustness of CG-DSF under challenging offshore inspection conditions, perturbation-based robustness tests were conducted on the test set. The perturbations were designed to simulate typical image degradation and sensor uncertainty during UAV-based blade inspection, including illumination variation, fog or low contrast, motion blur, thermal drift, and partial sensor degradation. These conditions do not change the ground-truth risk labels, but they reduce the quality of visible or infrared evidence and, therefore, test whether the model can maintain stable risk grading performance under degraded sensing conditions.

The results are summarized in Table 9. The original test condition corresponds to the standard paired visible–infrared samples without additional perturbation. For each degraded condition, the same trained model and the same risk grading thresholds were used, so that the performance change mainly reflects robustness to sensing degradation rather than retraining effects.

As shown in Table 9, CG-DSF maintains relatively stable performance under different degraded inspection conditions. Compared with the original condition, illumination variation, fog, rain, or low visibility, motion blur, and thermal drift all lead to performance degradation, but the decreases remain within an acceptable range. This indicates that the proposed consistency-guided fusion strategy can reduce the influence of local image degradation and sensor-specific interference to a certain extent.

Among the tested conditions, partial sensor degradation causes the largest performance decrease. This result is reasonable because severe degradation of one sensing channel weakens the complementary relationship between visible structural evidence and infrared thermal evidence. Nevertheless, CG-DSF still maintains higher performance than the single modality variants reported in Table 6, indicating that reliability-aware fusion can preserve useful information from the available sensor evidence rather than completely depending on one modality. These results support the robustness of CG-DSF under typical UAV inspection disturbances, while also showing that severe sensor degradation remains a challenging condition.

In addition to image degradation, visible infrared misalignment is another important issue in practical UAV inspection. Perfect pixel-level registration between visible-light frames and infrared thermal frames is difficult to guarantee because of differences in sensor field of view, imaging resolution, thermal diffusion, and UAV motion. Therefore, an additional misalignment test was conducted to evaluate whether CG-DSF can tolerate a moderate spatial mismatch between the two sensing modalities.

In this test, artificial shifts were applied to the infrared frame while the visible frame was kept unchanged. The shift levels were set as no additional shift, mild shift, moderate shift, and severe shift. Specifically, no additional shift denotes the original paired visible-infrared samples without extra translation. Mild shift was defined as a translation of 2% of the shorter image side. Moderate shift was defined as a translation of 5% of the shorter image side, and severe shift was defined as a translation of 10% of the shorter image side. For each shifted sample, the infrared frame was translated in a randomly selected horizontal and vertical direction, while the visible frame was kept unchanged. The same trained model and the same risk-grading thresholds were directly used for evaluation under all shift conditions. The results are reported in Table 10.

As shown in Table 10, mild visible–infrared misalignment only causes a small decrease in diagnostic performance. This indicates that the feature-level alignment module and local neighborhood compensation used in spatial consistency analysis can tolerate limited spatial mismatch between visible and infrared observations. Under moderate misalignment, the performance decreases more clearly but remains relatively stable, suggesting that CG-DSF does not rely on strict pixel-level registration.

However, severe misalignment leads to a more obvious performance drop. This result shows that, although feature-level regional alignment can reduce the influence of moderate mismatch, extremely inaccurate cross-sensor correspondence still weakens the reliability of consistency estimation. Therefore, CG-DSF should be used with basic frame-level pairing and quality screening in practical UAV inspection. More advanced registration or sensor synchronization strategies can be further incorporated in future work to improve robustness under severe misalignment. It should also be noted that the above artificial translation experiment is a controlled approximation of visible-infrared misalignment. It is useful for evaluating the sensitivity of CG-DSF to different degrees of spatial displacement, but it cannot fully replace real field misalignment. In practical UAV inspection, cross-sensor mismatch may also be caused by differences in sensor optical centers, field of view, lens distortion, rolling-shutter effects, UAV attitude changes, and nonlinear thermal diffusion. Therefore, the current synthetic shift test mainly provides a controlled robustness analysis. Future work will further evaluate CG-DSF on manually registered or naturally misaligned visible–infrared field samples when such paired data become available.

### 3.7. Wind-Farm Risk Distribution Case Study

To further illustrate the spatial representation of the risk diagnosis results produced by CG-DSF, a real satellite image of a wind turbine array is selected in this paper as the geographic reference scene, and the model output risk scores and risk levels are mapped onto the wind turbine array for case-based visualization analysis.

**Figure 9:**
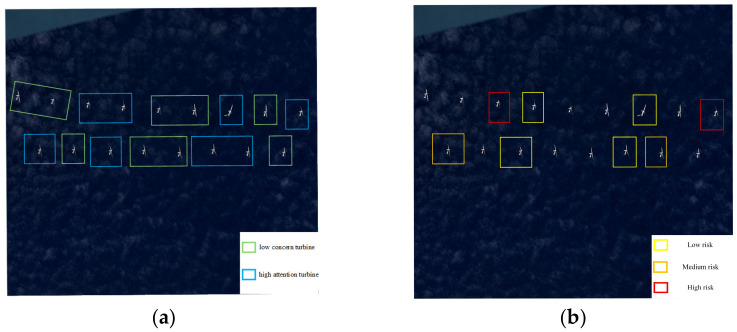
Case-based visualization analysis: (**a**) First-stage risk screening map of the wind turbine array. (**b**) Fine-grained risk stratification map based on CG-DSF diagnosis.

Figure 9a presents the array-level risk screening results in the first stage, where green boxes denote low attention wind turbines and blue boxes denote high attention wind turbines. It can be observed that all wind turbines are not simply assigned by the model to the same state. Instead, differentiated screening results are formed for different wind turbines according to the dual-spectrum diagnostic evidence. Low attention wind turbines are mainly distributed in positions with weaker risk responses in the array, whereas high attention wind turbines are concentrated in several local regions. This indicates that the risk scores output by CG-DSF can provide a preliminary screening effect at the array level and offer candidate objects for subsequent focused review.

Based on the first-stage screening results, the candidate wind turbines are further divided into different risk levels in the second stage, so that the risk diagnosis results can be refined, as shown in Figure 9b, where yellow boxes denote low risk, orange boxes denote medium risk, and red boxes denote high risk. It can be observed from the figure that most wind turbines remain in the low-risk or unmarked state, indicating that excessive warning is not issued by the model for the entire array. Medium-risk wind turbines are mainly distributed at certain positions requiring further observation, suggesting that some structural anomaly or thermal anomaly evidence may exist. The number of high-risk wind turbines is relatively small, but their locations are relatively clear, and they should be treated as priority targets for subsequent inspection review.

Overall, this case indicates that CG-DSF can provide not only the risk level of an individual sample but also spatial mapping of risk scores and grading results onto the wind turbine array, through which an intuitive risk distribution map can be formed. The first-stage screening facilitates rapid localization of wind turbines requiring attention, while the second-stage grading further distinguishes low risk, medium-risk, and high-risk objects. It should be noted that this case analysis is mainly intended to demonstrate the risk distribution representation capability of the model at the image diagnosis level. Final maintenance decisions should still be comprehensively determined by incorporating manual review, historical operating conditions, and on-site inspection results.

To illustrate the interpretability of the CG-DSF output, the main cross-sensor evidence and risk levels in the case are summarized in Table 11:

### 3.8. Complexity and Deployment Feasibility Analysis

To evaluate the computational cost and deployment feasibility of CG-DSF, the number of parameters, floating-point operations, average inference time, and frames per second were further analyzed. All deep learning models were evaluated using the same input size and the same hardware environment. The inference time was measured as the average processing time for one visible–infrared paired sample. For XGBoost, the inference time refers to the prediction time based on the organized diagnostic feature vector. The results are reported in Table 12. However, Params and FLOPs are not reported for XGBoost because it is a tree-based ensemble model operating on organized diagnostic features rather than a neural network processing raw dual-spectrum images.

As shown in Table 12, N/A indicates not applicable. Params and FLOPs are not reported for XGBoost because XGBoost is a tree-based ensemble model using organized diagnostic features rather than a neural network processing raw dual-spectrum images. XGBoost has the lowest inference time because it uses organized diagnostic features rather than raw dual-spectrum images, but its diagnostic performance is lower than that of deep learning models. Among the image-based models, the multimodal Transformer has a relatively high computational cost due to global attention-based multimodal interaction. In contrast, CG-DSF achieves a better balance between diagnostic performance and computational efficiency. Its parameters and FLOPs are lower than those of the multimodal Transformer, and its inference speed reaches 59.5 FPS under the tested hardware environment.

The efficiency of CG-DSF mainly benefits from lightweight convolutional branches and feature-token-level cross-sensor alignment. Since the alignment is performed on regional feature tokens rather than dense image pixels, the additional cost of consistency-guided fusion remains acceptable. These results indicate that CG-DSF is suitable for the offline batch analysis of UAV inspection data and near-real-time risk screening on an edge GPU or workstation. However, fully onboard real-time deployment on low-power UAV hardware still requires further model compression and hardware-aware optimization.

## 4. Discussion

Although the proposed CG-DSF framework achieves favorable performance in the dual-spectrum blade defect diagnosis task, certain limitations remain. Infrared thermal imaging results are susceptible to environmental and acquisition conditions, and complex environmental factors may alter the thermal response characteristics of blade surfaces. Although unstable thermal evidence is mitigated in this paper through infrared thermal image preprocessing, cross-sensor consistency analysis, and a reliability-oriented fusion strategy, the robustness of the model under complex marine weather, different seasons, and different inspection conditions still requires further verification.

The robustness and misalignment analyses further show that CG-DSF can maintain relatively stable performance under several simulated inspection disturbances and moderate visible–infrared mismatch. However, these tests are still based on the constructed paired sample subset from the public AQUADA-GO data. Therefore, the current results should not be interpreted as complete validation across all wind turbine types, UAV platforms, seasons, weather conditions, and unseen defect scenarios. In practical offshore inspection, environmental conditions, imaging distances, sensor calibration states, and defect manifestations may vary significantly across wind farms. Future work will further introduce multi-site data, different UAV imaging platforms, field-confirmed maintenance records, and cross-season inspection samples to evaluate the generalization capability of CG-DSF more comprehensively.

Despite these limitations, the proposed method remains meaningful for practical inspection scenarios. The main value of CG-DSF lies in the explicit modeling of spatial consistency, semantic consistency, anomaly consistency, and sensor reliability, so that whether the evidence from the two types of sensors mutually supports each other can be determined. Therefore, risk levels can be output by the model, and the formation mechanism of risk judgments can also be interpreted from the perspective of cross-sensor evidence.

Future research can be further conducted from three aspects. First, more real blade inspection samples, verified by field review, can be introduced to improve the engineering credibility of defect severity and risk-level annotations. Second, information such as turbine operating states, environmental conditions, and historical operation and maintenance records can be integrated to strengthen the association between image-based diagnosis results and actual blade health states. Third, cross-scenario validation can be carried out across different wind farms, seasons, turbine types, and inspection conditions to further evaluate the generalization capability and engineering applicability of CG-DSF.

## 5. Conclusions

To achieve reliable diagnosis and risk grading of offshore wind turbine blade defects under complex marine inspection environments, a defect diagnosis method based on cross-sensor consistency guided dual-spectrum imaging sensor fusion, denoted as CG-DSF, is proposed in this paper. Visible-light images and infrared thermal images are used as inputs. Through dual-branch feature extraction, cross-sensor alignment, and consistency-guided fusion, blade defect localization, identification, and risk grading are achieved. The main conclusions of this paper are as follows:

(1) Dual-spectrum diagnosis with visible-light images and infrared thermal images. CG-DSF simultaneously utilizes blade appearance structural evidence and thermal anomaly evidence. Through dual-branch modeling, sensor-specific feature extraction, and cross-sensor feature alignment, the insufficiency of diagnostic information from a single sensor under complex inspection conditions is compensated;
(2) Cross-sensor consistency-guided fusion. CG-DSF determines whether the evidence from visible-light images and infrared thermal images is mutually supportive from three aspects: spatial consistency, semantic consistency, and anomaly consistency. Combined with a reliability-oriented fusion strategy, the contributions of the two types of sensor features are adaptively adjusted;
(3) Systematic evaluation, robustness analysis, and engineering case validation. Through multi-metric comparison, ablation experiments, robustness and misalignment analyses, complexity evaluation, and wind turbine array case visualization, the advantages of CG-DSF in risk level diagnosis, key module effectiveness, sensing-condition robustness, deployment feasibility, and spatial representation of risk are verified.

## Figures and Tables

**Table 1:** Dataset statistics and risk-label distribution of the constructed paired-sample subset.

Subset	Number of Paired Samples	Low Risk	Medium Risk	High Risk
Training set	1440	544	496	400
Validation set	360	136	124	100
Test set	450	170	155	125
Total	2250	850	775	625

**Table 2:** AUROC comparison of different models.

Model	AUROC–Low Risk	AUROC–Medium Risk	AUROC–High Risk
CG-DSF	0.974	0.956	0.981
XGBoost	0.912	0.891	0.905
ViT	0.936	0.916	0.941
YOLOv8	0.925	0.901	0.934
Multimodal Transformer	0.951	0.932	0.963

**Table 3:** AUPRC comparison of different models.

Model	AUPRC–Low Risk	AUPRC–Medium Risk	AUPRC–High Risk
CG-DSF	0.962	0.935	0.958
XGBoost	0.858	0.831	0.846
ViT	0.902	0.874	0.911
YOLOv8	0.884	0.861	0.895
Multimodal Transformer	0.926	0.904	0.934

**Table 4:** Classification and ordinal risk-grading performance of different models.

Model	Accuracy	Weighted Kappa	Ordinal MAE	Macro F1
CG-DSF	0.924	0.908	0.080	0.923
XGBoost	0.871	0.838	0.140	0.870
ViT	0.893	0.867	0.116	0.892
YOLOv8	0.884	0.854	0.129	0.883
Multimodal Transformer	0.916	0.898	0.089	0.915

**Table 5:** Diagnostic accuracy across defect categories.

Defect Category	Precision	Recall	F1 Score
Crack	0.932	0.914	0.923
Leading-edge erosion	0.918	0.903	0.910
Coating damage	0.895	0.884	0.889
Thermal anomaly	0.926	0.897	0.911
Average	0.918	0.900	0.908

**Table 6:** Ablation results of different CG-DSF variants.

Model Variant	Accuracy	Macro F1	Macro AUROC	Macro AUPRC
CG-DSF-RGB	0.812	0.810	0.874	0.806
CG-DSF-IR	0.798	0.795	0.862	0.789
Direct Fusion	0.849	0.848	0.902	0.844
*w*/*o* Alignment	0.873	0.872	0.924	0.879
*w*/*o* SC	0.889	0.888	0.937	0.896
*w*/*o* SeC	0.897	0.896	0.943	0.907
*w*/*o* AC	0.884	0.883	0.935	0.891
*w*/*o* RAF	0.902	0.901	0.949	0.916
CG-DSF	0.924	0.924	0.970	0.952

**Table 7:** Risk-score distribution of different risk levels.

Risk Level	Mean Risk Score	Std.
Low risk	0.214	0.083
Medium risk	0.536	0.112
High risk	0.821	0.096

**Table 8:** Engineering-oriented inspection-priority evaluation under limited review resources.

Review Proportion	High-Risk Hit@k/Precision@k	Lift@k
Top 10%	0.846	3.05
Top 20%	0.742	2.67
Top 30%	0.631	2.27

**Table 9:** Robustness analysis under challenging inspection conditions.

Condition	Accuracy	Macro F1	Macro AUROC	Macro AUPRC
Original condition	0.924	0.924	0.970	0.952
Illumination variation	0.907	0.906	0.958	0.937
Fog, rain, or low visibility	0.895	0.894	0.949	0.925
Motion blur	0.889	0.888	0.944	0.918
Thermal drift	0.902	0.901	0.953	0.931
Partial sensor degradation	0.876	0.875	0.933	0.902

**Table 10:** Robustness analysis under visible infrared misalignment.

Misalignment Level	Accuracy	Macro F1	Macro AUROC	Macro AUPRC
No additional shift	0.924	0.924	0.970	0.952
Mild shift	0.913	0.912	0.962	0.941
Moderate shift	0.896	0.895	0.951	0.927
Severe shift	0.862	0.861	0.928	0.895

**Table 11:** Representative cross-sensor interpretation cases.

Case	Risk Level	Main Evidence	Cross-Sensor Consistency
Leading-edge erosion	Medium	Edge wear; Weak thermal response	Moderate spatial consistency; weak anomaly consistency
Surface crack	High	Crack trace; Thermal anomaly	High spatial consistency; high anomaly consistency
Coating damage	Low	Surface texture change; No thermal response	Weak anomaly consistency
Thermal anomaly	Medium	Weak visual change; Local heat response	Moderate semantic consistency; strong thermal evidence
Suspected delamination	High	Weak visual cue; Strong thermal discontinuity	High anomaly consistency with limited visual evidence

**Table 12:** Complexity and inference efficiency comparison of different models.

Model	Params (M)	FLOPs (G)	Inference Time (ms)	FPS
XGBoost	N/A	N/A	3.8	263.2
ViT	86.6	17.5	18.6	53.8
YOLOv8	11.2	28.6	14.3	69.9
Multimodal Transformer	72.4	24.8	24.1	41.5
CG-DSF	18.7	9.6	16.8	59.5

## Data Availability

The original optical and infrared wind turbine blade video data used in this study are publicly available from the AQUADA-GO project or its official data repository. The processed data, including paired visible and infrared samples, defect annotations, and risk-level labels generated in this study, are available from the corresponding author upon reasonable request.

## Abbreviations

The following abbreviations are used in this manuscript:

CG-DSF	Cross-Sensor Consistency-Guided Dual-Spectrum Imaging Sensor Fusion
RGB	Red, Green, and Blue
IR	Infrared
UAV	Unmanned Aerial Vehicle
AUROC	Area Under the Receiver Operating Characteristic Curve
AUPRC	Area Under the Precision Recall Curve
ROC	Receiver Operating Characteristic
PR	Precision Recall
IoU	Intersection over Union
ViT	Vision Transformer
YOLOv8	You Only Look Once version 8
SC	Spatial Consistency
SeC	Semantic Consistency
AC	Anomaly Consistency
RAF	Reliability Aware Fusion
